# Pleiotrophin, a target of miR‐384, promotes proliferation, metastasis and lipogenesis in HBV‐related hepatocellular carcinoma

**DOI:** 10.1111/jcmm.13213

**Published:** 2017-05-30

**Authors:** Pei‐song Bai, Nan Xia, Hong Sun, Ying Kong

**Affiliations:** ^1^ Department of Oncology First Hospital of Xi'an Jiaotong University Xi'an Shaanxi China; ^2^ Institute of Cancer Prevention and Control Peking University Cancer Hospital Bei'jing China

**Keywords:** hepatocellular carcinoma, hepatitis B virus, miR‐384, pleiotrophin, metastasis, lipogenesis

## Abstract

Hepatitis B virus (HBV) infection plays a crucial role and is a major cause of hepatocellular carcinoma (HCC) in China. microRNAs (miRNAs) have emerged as key players in hepatic steatosis and carcinogenesis. We found that down‐regulation of miR‐384 expression was a common event in HCC, especially HBV‐related HCC. However, the possible function of miR‐384 in HBV‐related HCC remains unclear. The oncogene pleiotrophin (PTN) was a target of miR‐384. HBx inhibited miR‐384, increasing PTN expression. The PTN receptor N‐syndecan was highly expressed in HCC. PTN induced by HBx acted as a growth factor *via* N‐syndecan on hepatocytes and further promoted cell proliferation, metastasis and lipogenesis. PTN up‐regulated sterol regulatory element‐binding protein 1c (SREBP‐1c) through the N‐syndecan/PI3K/Akt/mTORC1 pathway and the expression of lipogenic genes, including fatty acid synthesis (FAS). PTN‐mediated *de novo* lipid synthesis played an important role in HCC proliferation and metastasis. PI3K/AKT and an mTORC1 inhibitor diminished PTN‐induced proliferation, metastasis and lipogenesis. Taken together, these data strongly suggest that the dysregulation of miR‐384 could play a crucial role in HBV related to HCC, and the target gene of miR‐384, PTN, represents a new potential therapeutic target for the prevention of hepatic steatosis and further progression to HCC after chronic HBV infection.

## Introduction

HCC is one of the most common human malignancies in China and has a poor prognosis and low survival rate 1. Thus, it is a very serious health problem. Chronic infection with HBV is a major risk factor for the development of HCC 2. The mechanism by which HBV induces the events leading to HCC has not been fully elucidated. Hepatitis B virus X protein (HBx), a multifunctional transactivator protein, is considered one of the most important determinants of HBV‐induced hepatocarcinogenesis and acts as an oncogene or cofactor 3, 4.

In addition, abnormal hepatic lipogenesis frequently occurs in chronic HBV‐infected patients 5. In normal tissues, lipids are derived from circulating lipids, while most cancer cells do not use fatty acids (FAs) from the circulation but mainly synthesize lipids *de novo* 6. *De novo* lipogenesis plays an important role in tumour development and is increasingly recognized as an important hallmark of cancer 6. Highly proliferative tumour cells must synthesize *de novo* FAs to supply lipid signalling molecules, post‐translational protein modifications and membrane building blocks to support rapid cell proliferation and metastasis 7. However, the mechanisms underlying these phenomena are not fully understood. The increased *de novo* FAS in cancer cells occurs through multiple mechanisms, most of which involve the abnormal expression of key lipogenic enzymes and miRNAs. miRNAs play an important role in HBV‐induced lipid metabolism disorders and HBV‐related liver diseases 8, 9.

Our results showed that miR‐384 was down‐regulated in HBV‐related HCC, and that the expression of miR‐384 was negatively correlated with HBV infection. The low expression level of miR‐384 was found to predict a poor prognosis in HCC patients. MiR‐384 inhibits cell proliferation, colony formation and metastasis *in vitro*. We scrutinized the target genes of miR‐384 using bioinformatics methods. We found that miR‐384 was capable of down‐regulating PTN in hepatoma cells by directly binding to its 3′UTR. The oncogene PTN is a strong hepatocyte mitogen and is associated with liver regeneration. Increased PTN expression has been reported in multiple tumour types. PTN expression was significantly higher in tumour samples than in control samples, which was especially evident in HBV‐positive tumour tissues, and the inhibition of HBV replication could down‐regulate the expression of PTN. PTN could promote proliferation and metastasis *in vitro*. Increased PTN expression could promote tumour proliferation and angiogenesis *in vivo*. The PTN receptor N‐syndecan was found to be highly expressed in HCC. The expression of N‐syndecan was positively correlated with HBV infection. HBV could up‐regulate N‐syndecan expression. PTN acts as a growth factor *via* N‐syndecan on hepatoma cells to promote cell proliferation, metastasis and lipogenesis. Up‐regulation of lipid synthesis in HCC has been identified as a crucial step to overcoming metabolic stress for cancer cell survival and metastasis 10. However, little is known regarding whether PTN is involved in the lipogenesis of hepatoma cells induced by HBV.

Our results showed that PTN is an important regulator of lipogenesis and can regulate genes encoding proteins associated with *de novo* lipogenesis in tumour cells. In particular, SREBP‐1c, the major transcriptional factor involved in regulating FAS, is an important downstream target of PTN. Our findings demonstrate that PTN promotes *de novo* lipogenesis of hepatoma cells by up‐regulating the lipogenic enzymes FAS *via* the N‐syndecan/PI3K/Akt/mTORC1/SREBP‐1c signalling pathway. Proliferation, angiogenesis and *de novo* lipogenesis in hepatoma cells regulated by PTN are important for the progression of HCC.

In summary, we studied the effects of HBx on hepatocarcinogenesis and hepatoma cell lipogenesis. Taken together, the above findings indicate that dysregulation of miR‐384 may play an important role in HBV‐induced HCC. HBx inhibits miR‐384, which results in PTN overexpression to promote proliferation, metastasis and lipogenesis in hepatoma cells.

## Materials and methods

### Tissue samples and clinical data

Tissue samples were collected from 80 HCC patients who underwent routine curative surgery, including samples of adjacent non‐cancerous liver tissue (not less than 2 cm from the margin of resection, and pathologically confirmed) from January 2007 to December 2012. The median follow‐up period was 36.3 months. The tissues were flash frozen in liquid nitrogen or fixed in formalin for histological and immunohistochemical analyses. The tissue samples were used after obtaining informed consent from every patient. The clinicopathological characteristics of all patients are presented in Table 1. None of the samples had been pre‐treated with any chemotherapy or embolization prior to surgery. Twenty normal liver tissue samples were obtained from healthy living transplant donors. Fifty‐five (68.75%) patients had died, including 16 who died from liver failure, gastrointestinal bleeding and bacterial infection and 39 who died due to tumour recurrence. The protocol was approved by the Human Ethics Committee at the First Affiliated Hospital of Xi'an Jiaotong University. All participants provided informed consent prior to their inclusion in the study. The ages and genders of the patients, as well as information regarding tumour stage, tumour differentiation and histopathological factors, were collected from the patient medical records at the First Affiliated Hospital of Xi'an Jiaotong University.

**Table 1:** Correlation between the clinicopathological characteristics and miR‐384 and PTN expression in HCC

Pathological factors	*n*	No. of patients			No. of patients		
		miR‐384	miR‐384	*P*	PTN	PTN	*P*
		Low	High		Negative (−, ±)	Positive (+, ++)	
Total no. of patients	80	51	29		33	47	
Age (years)							
<50	32	18	14	>0.05	13	19	>0.05
≥50	48	33	15		20	28	
Sex							
Male	61	39	22	>0.05	25	36	>0.05
Female	19	12	7		8	11	
HBV							
Absent	17	7	10	<0.05^a^	11	6	<0.05^a^
Present	63	44	19		22	41	
Tumour size (cm)							
<5	28	15	13	>0.05	12	16	>0.05
≥5	52	36	16		21	31	
Cirrhosis							
Absent	23	12	11	>0.05	14	9	<0.05^a^
Present	57	39	18		19	38	
Adjacent organ invasion							
Absent	60	34	26	<0.05^a^	29	31	<0.05^a^
Present	20	17	3		4	16	
Microscopic vascular invasion							
Absent	57	32	25	<0.05^a^	28	29	<0.05^a^
Present	23	19	4		5	18	
PVTT							
Absent	65	43	22	>0.05	27	38	>0.05
Present	15	8	7		6	9	
TNM tumour stage							
I + II	55	32	27	<0.05^a^	30	25	<0.05^a^
III + IV	25	23	2		3	22	
AFP							
≤400	49	33	16	>0.05	17	32	>0.05
>400	31	18	13		16	15	

HCC: hepatocellular carcinoma; HBV: hepatitis B virus; TNM: tumour node metastasis; PVTT: portal vein tumour thrombus; ‘−‘and ‘±’, negative; ‘+’ and ‘++’, positive.

a *P* < 0.05 was considered statistically significant.

### Collection of blood samples

We collected blood samples from 15 healthy volunteers, 25 patients with HBV‐related hepatitis, 17 patients with HBV‐related cirrhosis, 20 patients with HBV‐related HCC and 11 patients with non‐HBV‐related HCC. Twenty patients with HBV‐related HCC received entecavir for antiviral treatment. When their levels of hepatitis B DNA were less than 1.0 e + 003 IU/ml, new blood samples were collected.

We collected 17 blood samples from HCC patients who were treated with transcatheter hepatic arterial chemoembolization (TACE) pre‐operatively and post‐operatively for 2, 14 and 28 days.

Five millilitres of blood (non‐anticoagulated) was collected from patients who had fasted the previous night. These blood samples were allowed to stand for 30 min. and were then centrifuged at 4°C and 2683 g for 5 min. Fresh serum was separated and stored at −80°C; hemolysed cells and the buffy coat were discarded.

### Vector construction and transfection

Stable cell transfection to up‐regulate PTN and HBx expression was performed as previously described. Briefly, PTN (NM_002825.5) and HBx (AY839630.1) were inserted into the pcDNA3.1 (+) mammalian expression vector (Invitrogen, Carlsbad, CA, USA). A PTN shRNA plasmid (psiHIV‐U6‐shRNA) and scrambled non‐target negative control were obtained from the OmicsLink™ shRNA Expression Clone Datasheet of GeneCopoeia, Inc. Cells were seeded in DMEM containing 10% foetal bovine serum (Gibco, Carlsbad, CA, USA). Twenty‐four hours later, the cells were transfected with Transfast™ Transfection Reagent (Promega, Madison, WI, USA), according to the manufacturer's instructions. The pcDNA3.1 (+) expression vector and the scrambled non‐target shRNA plasmid were used as negative controls.

A lentiviral packaging kit was purchased from Open Biosystems (Huntsville, AL, USA). Lentiviruses carrying miR‐384 or a miR‐negative control (miR‐NC) were packaged according to the manufacturer's instructions.

### Cell culture

The human HCC cell lines HepG2 and Huh‐7 (human hepatoma cells expressing PTN) and the human LX‐2 (hepatic stellate cells, HSCs) cell line and human normal liver cell line LO2 were purchased from the Institute of Biochemistry and Cell Biology of the Chinese Academy of Sciences (Shanghai, China). HepG2.2.15 (a hepatoma HepG2 cell line stably transfected with full‐length HBV genomes) was a gift from the Department of Hepatobiliary Surgery, First Hospital of Xi'an Jiaotong University. HepG2.2.15 could secrete complete HBV virus particles into the culture supernatant *in vitro* and also produce a large number of replication intermediates such as HBx. In our study, we collected HBV virus particles from the supernatant of cultured HepG2.2.15 cells and measured the titre of HBV virus particles. These samples were used as a source of infection to infect hepatocytes. The cells were cultured in DMEM containing 10% foetal bovine serum (Gibco) (pH 7.4) at 37°C in a humidified atmosphere with 5% CO_2_, and the culture medium was changed every 2–3 days. All cells used in the experiments were in the log‐growth phase. The cells were treated with 10 μm LY294002 (Sigma‐Aldrich, St. Louis, MO, USA) and 50 nm rapamycin (Sigma‐Aldrich).

### PTN ELISA

The samples were analysed by ELISA as previously described. Briefly, patient serum samples were incubated overnight at 4°C in 96‐well microtitre plates. The plates were washed three times with PBS and blocked with PBS containing 0.1% Tween‐20 (PBST) supplemented with 1% bovine serum albumin at room temperature for 2 hrs. The plates were then incubated with 100‐μl/well biotinylated anti‐PTN at 4°C overnight, washed three times with PBST, incubated with 100 μl of alkaline phosphatase‐conjugated streptavidin (AnaSpec, Fremont, CA, USA) at room temperature for 1 hr (200 mg/ml, 1:200) and washed four times with PBST. The results were analysed using the mQuant microplate reader (BioTek Instruments, Inc., Winooski, VT, USA) at 450 nm, and the concentrations were calculated using microplate reader software (BioTek Instruments, Inc.).

### Immunohistochemical staining

Immunohistochemistry was performed using 5‐μm‐thick serial sections derived from formaldehyde‐fixed, paraffin wax‐embedded tumour tissue blocks. After deparaffinization and rehydration, endogenous peroxidase activity was inactivated with 0.3% H_2_O_2_ in methanol for 30 min., and endogenous biotin was blocked with a biotin blocking kit (Vector Laboratories, Burlingame, CA, USA), according to the manufacturer's instructions. All staining steps were completed at room temperature, and the samples were washed with PBS between steps. Sections were dewaxed in three different concentrations of xylene for 5 min. and heated in a microwave to 98°C for 15 min. in citric acid buffer (pH 6.0). These sections were then incubated overnight at 4°C in a humidified chamber with goat polyclonal anti‐PTN antibodies [Santa Cruz (sc‐1394) goat polyclonal antibody]. Biotinylated secondary antibodies (Santa Cruz Biotechnology, Santa Cruz, CA, USA) were applied for an additional hour following removal of the primary antibodies. Staining was performed with an avidin‐biotinylated horseradish peroxidase complex and diaminobenzidine (Santa Cruz Biotechnology), according to the manufacturer's directions. The primary antibody was replaced with either PBS or normal serum from the same species, which served as a negative control. The supernatant from the reaction mixture did not show any immunoreactivity in the liver tissues when used for immunohistochemistry, supporting the reliability and validity of the antibody for the immunohistochemical analysis performed in this study. Scoring criteria: samples were considered negative if less than 5% of cells were stained with PTN; weak‐positive (±) with 5–25% staining; positive (+) with 25–50% staining; strongly positive (+ +) with positive cytoplasmic staining of more than 50% of cells.

### RNA isolation and endogenous miR‐384 expression assay

RNA isolation and RT‐PCR were performed as previously described. Total RNA, including total miRNA, was isolated from the cultured cell lines and tissue samples using a Qiazol and miRNeasy Mini Kit (Qiagen, Germantown, MD, USA), according to the manufacturer's instructions. The final volume of RNA (total RNA containing microRNA), which was stored at −80°C, was 50 μl. The RNA purity and integrity were analysed using an Agilent Bioanalyzer 2100 (Agilent Technologies, Santa Clara, CA, USA). The cDNAs were synthesized using a PrimeScript™ 1st Strand cDNA Synthesis Kit (Invitrogen). The relative amount of mRNA was determined with gene‐specific primers. All steps were performed according to the manufacturer's protocol. The primers were as follows: PTN (NM_002825.5), 5′‐ACCAGTGAGTCATCCGTCCA‐3′ (forward) and 5′‐TGCAAATTTTCGACGCTGCT‐3′ (reverse); HBx (AY839630.1), 5′‐TCTGTGCCTTCTCATCTGC‐3′ (forward) and 5′‐TCGGTCGTTGACATTGCTG‐3′ (reverse); N‐syndecan(NM_014654.3), 5′‐ACGCGTCCTTCCAAGAATGT‐3′(forward) and 5′‐TGTTCTGGCTCGATTCCACC‐3′(reverse); and β‐actin (NM_001101.3), 5′‐CTCACCATGGATGATGATATCGC‐3′ (forward) and 5′‐AGGAATCCTTCTGACCCATGC‐3′ (reverse).

The expression levels of miR‐384 in serum and tissues were quantified with an miScript SYBR Green PCR Kit (Qiagen) using a miRNA‐specific forward primer and a universal poly(T) adaptor reverse primer, according to the manufacturer's instructions. All reactions were performed in duplicate. The relative amount of miR‐384 was normalized to the amount of U6. The level of miRNA expression was measured using the 2^−ΔDeltaCt^ method.

### miRNA target prediction and assay

Using four computer algorithms, including TargetScan, miRanda, PicTar and miRGen, we identified PTN as a possible miR‐384 target. The sites were predicted based on the base‐pairings of seed‐sequence matches. To construct reporter plasmids containing wild‐type or mutant miR‐384 target sites for human PTN 3′UTR segments, oligonucleotide pairs containing the desired miR‐384 target region and restriction enzymes sites were annealed and ligated into the pMIR‐REPORT™ miRNA Expression Reporter Vector (Ambion, Grand Island NY, USA). The mutated putative miR‐384 binding site in the PTN 3′UTR was generated using a QuikChange site‐directed mutagenesis kit (Stratagene, La Jolla, CA, USA) according to the manufacturer's protocol. These cells were plated in 12‐well plates (3 × 10^4^ cells/well). Twenty‐four hours later, the pMIR‐PTN‐3′‐UTR construct (200 ng) and the β‐gal expression vector pMIR‐REPORT‐β‐gal (200 ng) (Ambion) were cotransfected with a miR‐384 mimic or a mimic control at a final concentration of 100 nM using Transfast™ Transfection Reagent (Promega). Twenty‐four hours after transfection, cell lysates were collected, and luciferase activity was measured according to the manufacturer's protocol. Firefly luciferase activity was normalized to β‐gal expression for each sample.

### Western blot analysis

Total soluble proteins (100 μg) extracted from the samples were resolved on 10% sodium dodecyl sulphate–polyacrylamide gels and transferred electrophoretically to a polyvinylidene fluoride membrane. The blots were blocked with 5% skim milk, followed by incubation with antibodies specific for PTN (C‐19) [Santa Cruz (sc‐1394) goat polyclonal antibody], SREBP‐1c [Abcam (ab3259) mouse monoclonal antibody], FAS [Santa Cruz (sc‐55580) mouse monoclonal antibody], phospho‐Akt (pSer473) [Cell Signaling Technology (#9271) mouse Polyclonal antibody], Akt (pan) (C67E7) [Cell Signaling Technology (#4691) rabbit monoclonal antibody], mTORC1 (7C10) [Cell Signaling Technology (#2983) rabbit monoclonal antibody] and phospho‐mTORC1 (Ser2448) [Cell Signaling Technology (#5536) rabbit monoclonal antibody]. Actin (I‐19) [Santa Cruz (sc‐1616) goat polyclonal antibody] was used as a loading control. The blots were then incubated with peroxidase‐labelled rabbit, mouse and goat secondary antibodies (1:2000, 1:3000 and 1:2000, respectively, Santa Cruz) and visualized using enhanced chemiluminescence.

### Cell proliferation, colony formation assay and cell invasion assay

To measure the effect of miR‐384 and PTN on cell proliferation, cells were seeded in 96‐well plates in triplicate at densities of 1 × 10^3^ per well. The plates were harvested for measurement at the indicated time points, and cell proliferation was assessed by the MTT assay using an assay kit (CellTiter 96 AQueous; Promega) according to the manufacturer's protocol. In brief, the MTT assay was performed by the addition of 10 μl MTT (10 mg/ml) for 4 hrs. Light absorbance of the solution was measured at 490 nm using a microtiter plate reader (Molecular Devices, Sunnyvale, CA, USA).

To assess the effect of miR‐384 and PTN on cell colony formation, a total of 1.0 × 10^5^ transfected and control cells were resuspended and seeded into a 6‐cm dish and maintained in DMEM containing 10% FBS for 2 weeks. The colonies were fixed with methanol and stained with 0.1% crystal violet for 15 min., and then they were counted using a light microscope (Olympus, Tokyo, Japan).

The cells were examined for their invasive ability *in vitro* in BD BioCoat Matrigel chambers (Transwell; BD Biosciences, San Jose, CA, USA). Briefly, Huh‐7‐miR‐384, Huh‐7‐anti‐miR‐384 cells, Huh‐7‐PTN and NC were seeded into the top chamber with a Matrigel‐coated filter and 750 μl of DMEM containing 5% foetal bovine serum as a chemoattractant. After incubation for 24 hrs at 37°C, non‐invading cells on the upper surface of the filter were removed with cotton swabs, and invading cells on the lower membrane surface were fixed and then stained with 5% crystal violet (Sigma‐Aldrich). The number of cells that had invaded through the Matrigel was normalized to those that traversed the non‐Matrigel to obtain an index of relative per cent invasion, indicating the ability of tumour cells to invade *in vitro*. Cells were counted in five fields for triplicate membranes at 10× magnification. Each condition was assayed in triplicate wells. The cells were imaged by phase‐contrast microscopy (Leica Microsystems, Bannockburn, IL, USA).

For the wound‐healing assays, the cells were plated in a 12‐well plate and cultured at 37°C for 24 hrs. Wounds were created in monolayers of cells using a 10‐μl pipette tip. The cells were washed to remove cellular debris and incubated in DMEM without FBS at 37°C. Images were obtained at different time points following wounding. The wound area was measured, and the percentage of wound healing was calculated using ImageJ software (NIH, Bethesda, MD, USA).

### *In vivo* assays for tumour growth

Huh‐7 cells (5 × 10^6^) transfected with miR‐384, PTN and control were implanted subcutaneously into the flank of nude mice (male BALB/c nu/nu, 4–6 weeks old) (Institute of Materia Medica, CAS, Shanghai, China), and tumour growth was monitored based on the tumour volume, which was calculated as follows: V (mm^3^) = width^2^(mm^2^) × length (mm)/2. The mice were killed 45 days later, and tumour nodes were removed. Consecutive sections were generated for every tissue block and stained with haematoxylin–eosin. Immunohistochemical staining was conducted with CD34 antibody [EP373Y] [Abcam (ab81289) rabbit monoclonal antibody] to analyse the microvessel density in tumour nodes. All procedures were in accordance with the Guide for the Care and Use of Laboratory Animals and the Institutional Ethical Guidelines for Animal Experiments. These procedures were approved by the Animal Care and Use Committee of Xi'an Jiaotong University, China.

### Oil Red O staining

Oil Red O (Sigma‐Aldrich) stock solution was prepared in isopropanol (0.5 g/100 ml) and heated to 100°C for 10 min. The cells were fixed with 4% paraformaldehyde for 30 min. and washed with PBS. After being washed with distilled water and 60% isopropanol, the cells were stained for 30 min. at room temperature with freshly prepared Oil Red O solution (0.2% Oil Red O in 60% isopropanol). These cells were then washed with PBS until their background became clear and were photographed. Oil Red O was eluted with 100% isopropanol and quantified by measuring the optical absorbance at 520 nm.

### Quantification of fatty acids

The cells were plated in 60‐mm culture dishes and cultured in DMEM containing 10% foetal bovine serum (Gibco) (pH 7.4) at 37°C in a humidified atmosphere with 5% CO_2_. The culture medium was changed every 2–3 days. Cell culture medium was replaced with DMEM without serum for 24 hrs. The cells were washed twice with cold PBS, lysed in PBS containing 1% Triton X‐100 for 30 min. at 4°C, and centrifuged at 10,000 × *g* for 10 min. at 4°C. Intracellular FA accumulation was evaluated after cell lysis. The triglyceride levels were measured using a Free Fatty Acid Quantification Kit (ab65341; Abcam, Cambridge, MA, USA) according to the manufacturer's instructions.

### Statistical analysis

The results are representative of three independent experiments and were analysed using SPSS, version 18.0 software (SPSS, Chicago, IL, USA) and GraphPad Prism 5 (GraphPad Software, Inc., San Diego, CA, USA). Statistical significance was defined as *P* < 0.05.

## Results

### MiR‐384 is down‐regulated in HCC tissues

**Figure 1:**
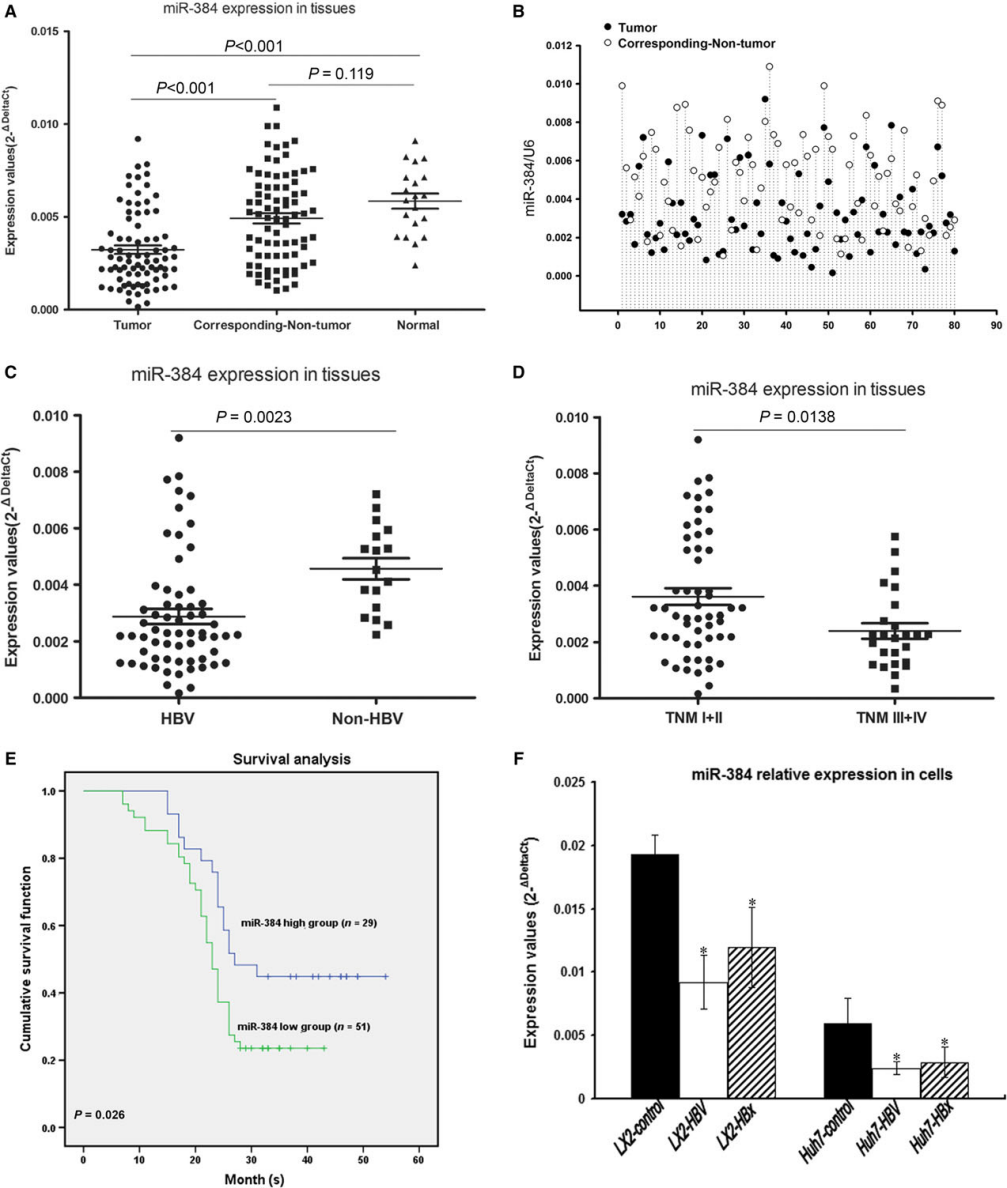
HBx inhibits miR‐384 expression in HBV‐related HCC. (**A**) Analysis of 80 paired tumour tissue samples, adjacent non‐tumour tissue samples and 20 normal tissue samples showing that the expression of miR‐384 was decreased in tumour tissues compared with non‐tumour tissues and normal tissues. (**B**) The expression of miR‐384 was decreased in 63.75% (51/80) HCC tissue samples compared with their adjacent normal controls. (**C**) HBV‐infected HCC patients exhibited lower expression levels of miR‐384 than HBV‐negative HCC patients. (**D**) HCC patients were divided according to tumour stage. The expression of miR‐384 was lower in patients with TNM III and IV than TNM I and II. (**E**) Kaplan–Meier survival curves in 80 patients with HCC according to the miR‐384 expression status. The results showed that low miR‐384 expression was predictive of a poor prognosis in HCC patients. The log‐rank test suggested that the difference was significant between the low miR‐384 expression group and the high miR‐384 expression group (*P* = 0.026). (**F**) miR‐384 expression was down‐regulated in HBV‐infected cells and HBx‐transfected cells compared with control cells. *P value <0.05.

We observed a down‐regulation of miR‐384 expression in tumour tissue compared with non‐tumour tissue and normal liver tissue (*P* < 0.05; Fig. 1A). The expression of miR‐384 was decreased in 63.75% (51/80) HCC tissue samples compared with their adjacent normal controls (Fig. 1B). Interestingly, HBV‐infected HCC patients showed lower expression levels of miR‐384 than HBV negative‐infected HCC patients (Fig. 1C), and the expression of miR‐384 was lower in patients with TNM III and IV than TNM I and II (Fig. 1D). As shown in Table 1, the decreased level of miR‐384 was significantly correlated with HBV infection, adjacent organ invasion, microscopic vascular invasion and advanced TNM tumour stage (*P* <  0.05). These results suggested that miR‐384 was down‐regulated in HBV‐related HCC, and that the expression of miR‐384 was negatively correlated with HBV infection. The aberrant expression of miR‐384 was correlated with poor clinical features of HCC patients. Thus, miR‐384 may function as a tumour suppressor.

### Low expression levels of miR‐384 resulted in a poor prognosis in HCC patients

A follow‐up was conducted to estimate the overall survival rate of HCC patients. Among the HCC patients, 51 were in the low miR‐384 expression group, and the rest were in the high miR‐384 expression group. Kaplan–Meier analysis showed that HCC patients with high levels of miR‐384 expression presented significantly longer survival times than those with low miR‐384 expression (χ^**2**^ **=** 4.979, *P* = 0.026; Fig. 1E).

### HBx inhibits miR‐384 expression and up‐regulates PTN expression

**Figure 2:**
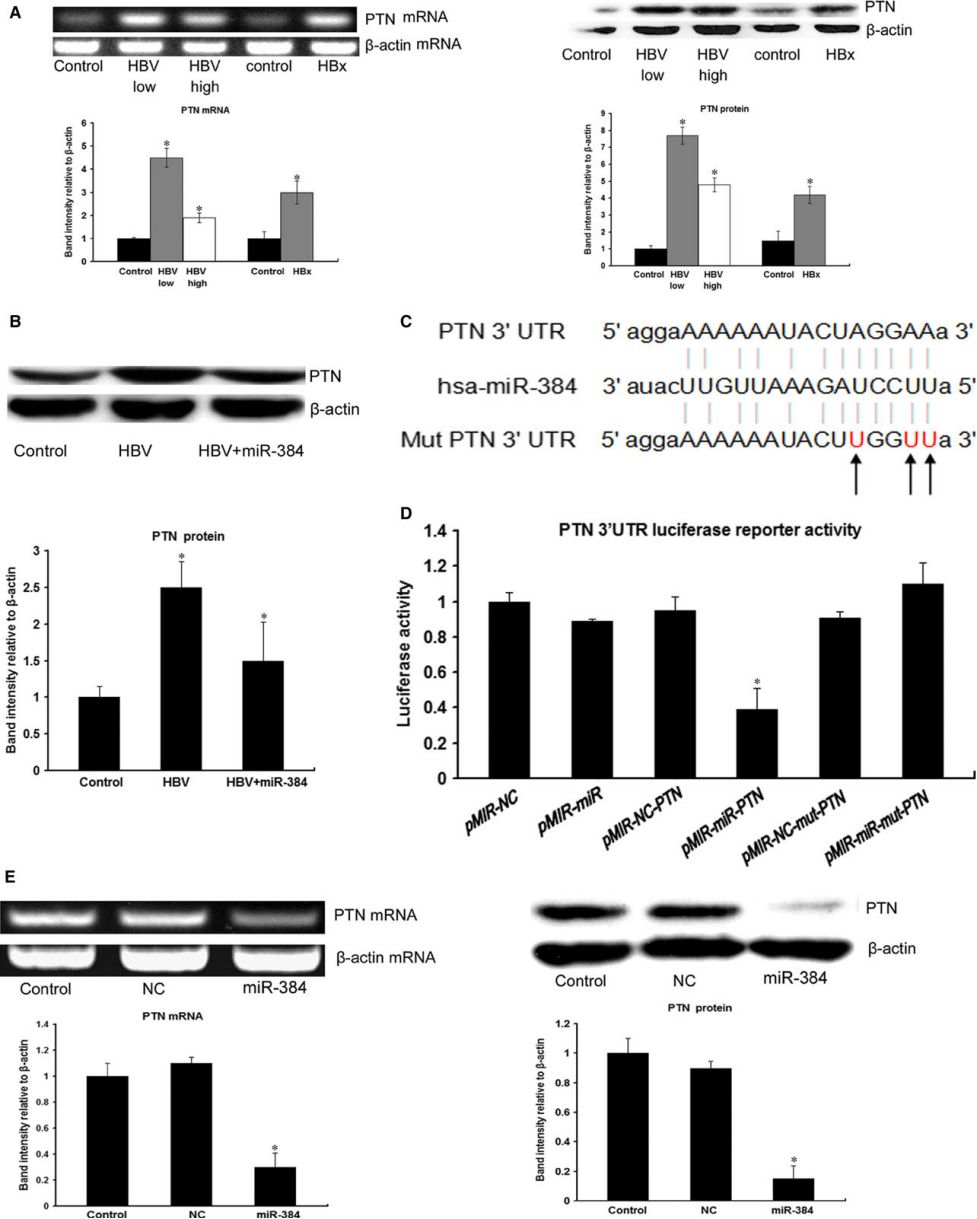
HBx up‐regulates PTN expression, and PTN is a target of miR‐384. (**A**) The expression of PTN was up‐regulated in HBV‐infected cells and HBx‐transfected cells compared with control cells. Our results indicated that the expression level of PTN was much higher in cells with low concentrations of HBV particles (1.0 × 10^3^ copies/ml) than in cells with high concentrations of HBV particles (1.0 × 10^5^ copies/ml). (**B**) The expression of PTN was up‐regulated in HBV‐infected cells compared with control cells. The expression of PTN was down‐regulated in HBV‐infected Huh‐7‐miR‐384 cells compared with HBV‐infected Huh‐7 cells. (**C**) The seed sequence of miR‐384 matched the 3′UTR of the PTN gene. The sequences shown include the mutated target mRNA site, the mature miR‐384 and the target mRNA site bound by miR‐384. (**D**) Luciferase assays of Huh‐7 cells transfected with the PTN reporter plasmid containing wild‐type (WT) regulatory sequences, controls (NCs), the miR‐384 precursor or the mutated PTN reporter (mut). We found that PTN 3′UTR luciferase reporter activity was regulated by miR‐384 cotransfection. The luciferase assay demonstrated that PTN was a direct target of miR‐384. (**E**) PCR and Western blotting of Huh‐7 cells transfected with the miRNA negative control (NC) and miR‐384. The results demonstrated that miR‐384 repressed PTN expression. *P value <0.05.

We cocultured HepG2.2.15 cells with LX‐2 and Huh‐7 cells or transfected HBx into LX‐2 and Huh‐7 cells. The results showed that miR‐384 expression was down‐regulated in HBV‐infected and HBx‐transfected compared with control cells (Fig. 1F), and that the expression of PTN was up‐regulated in HBV‐infected and HBx‐transfected compared with control cells (Fig. 2A). Our results indicated that the expression levels of PTN were higher in cells treated with low HBV particle concentrations (1.0 × 10^3^ copies/ml) than in cells treated with high HBV particle concentrations (1.0 × 10^5^ copies/ml) (Fig. 2A). The expression of PTN was up‐regulated in HBV‐infected cells compared with control cells. The expression of PTN was down‐regulated in HBV‐infected Huh‐7‐miR‐384 cells compared with HBV‐infected Huh‐7 cells (Fig. 2B). This finding suggested that HBV inhibited miR‐384 expression and up‐regulated PTN expression.

### PTN is a direct target of miR‐384

To determine whether PTN expression is regulated by miR‐384, we constructed luciferase reporter genes with PTN 3′UTRs with or without mutations in the miR‐384 binding regions and tested their expression after they were cotransfected into Huh‐7 cells with either miR‐384 mimics or miR‐NCs (Fig. 2C). Compared with the control, a decrease in relative luciferase activity was noted when the PTN 3′UTR was cotransfected with miR‐384. However, the miR‐384 mimic did not affect luciferase activity in the mutant construct; thus, there was no significant difference in luciferase activity between the mutant and the control (Fig. 2D). Furthermore, up‐regulation of miR‐384 resulted in a significant decrease in PTN expression in Huh‐7 cells (Fig. 2E). These data indicate that miR‐384 directly modulated PTN expression by binding to the 3′UTR.

### PTN expression was up‐regulated in human HCC tissue and serum and was inversely correlated with miR‐384 expression

**Figure 3:**
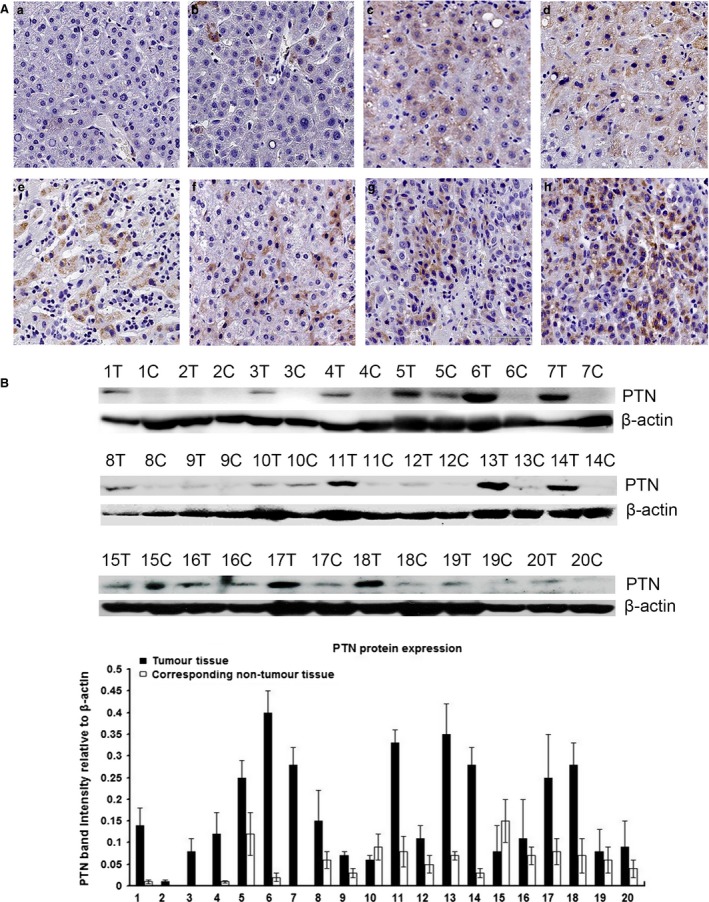
Analysis of the expression level of PTN in tissue. (**A**) PTN expression was much higher in HCC tissues than in non‐neoplastic and normal tissues. The IHC results showed that PTN was expressed in HSCs, hepatocytes and hepatoma cells, PTN protein in HCC was localized in the cytoplasm and the expression of PTN was elevated in some patients with steatosis (a: normal hepatic tissues; b: matched non‐neoplastic tissues; c and d: hepatitis tissues with partial steatosis; e and f: hepatic cirrhosis tissues; g and h: HCC tissues). (**B**) Western blot results showing that the expression of PTN was significantly higher in HCC tissues than in matched non‐neoplastic tissues.

We examined the expression levels of PTN in HCC specimens and serum samples *via* IHC, Western blot and ELISA. As shown in Figure 3A and B, the expression of PTN was significantly higher in HCC tissues than in matched non‐neoplastic tissues and normal tissues. The IHC results showed that PTN was expressed in HSCs, hepatocytes and hepatoma cells, PTN protein in HCC was localized in the cytoplasm, and the expression of PTN was elevated in some patients with steatosis (Fig. 3A). Statistical analysis showed that increased expression of PTN was correlated with HBV infection, cirrhosis, adjacent organ invasion, microscopic vascular invasion and advanced TNM stage (*P* < 0.05; summarized in Table 1). PTN was significantly increased in 47 of 80 (58.75%) tumour tissue samples compared with 15 of 80 (18.75%) matched non‐neoplastic tissue samples and 2 of 20 (10%) normal tissue samples (*P* < 0.05; Table 2).

**Table 2:** Comparative analysis of the expression of PTN in HCC tissues, matched non‐neoplastic tissues and normal tissues

Pathological factors	*N*	PTN expression (*n*, %)			χ^2^
		Positive	Negative	%	
HCC	80	47	33	58.57%	5.94
MNT	80	15	65	18.75%	*P* < 0.05^a^
Normal	20	2	18	10.0%	

a *P* < 0.05 was considered statistically significant.

MNT: Matched non‐neoplastic tissues; HCC: hepatocellular carcinoma.

**Figure 4:**
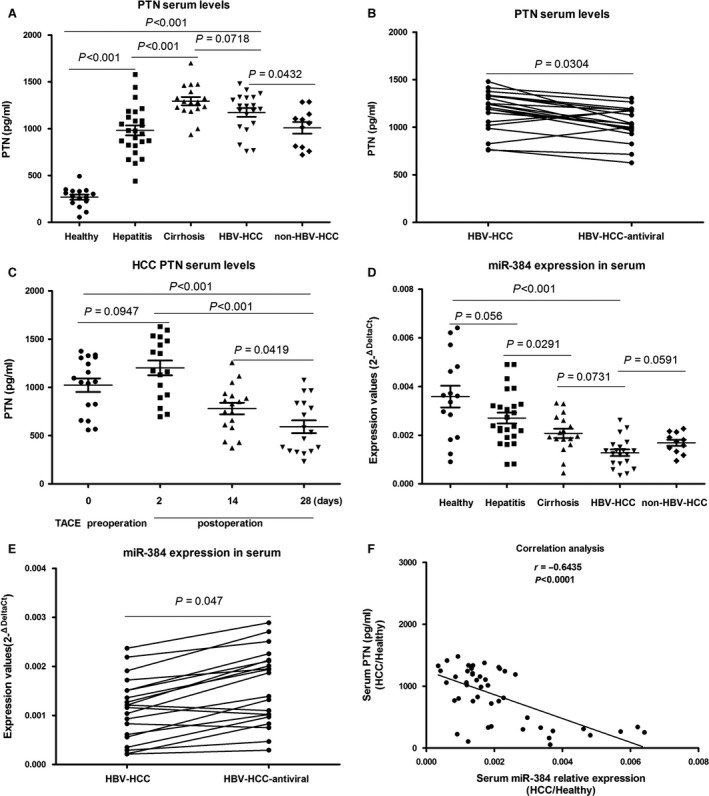
Analysis of the expression level of PTN and miR‐384 in serum samples. (**A**) Serum PTN expression was higher in HCC tissues than in healthy volunteers, and PTN expression was also higher in hepatitis tissues and hepatic cirrhosis tissues samples than in normal liver samples. PTN expression was highest in HBV‐related cirrhosis patients, and it was higher in HBV‐related HCC patients than in non‐HBV‐related HCC patients (*P* < 0.05). (**B**) The ability of hepatitis B virus replication to down‐regulate the expression of PTN (**C**) The serum PTN level rose slightly in 2‐day post‐operative blood samples compared with the pre‐operative blood samples (*P* > 0.05), but there was a gradual decline in the 14 and 28‐day postoperative blood samples. (**D**) Serum miR‐384 levels were significantly lower in tumour samples than in control samples (*P* < 0.05). Serum miR‐384 levels were lower in HBV‐related HCC patients than in non‐HBV‐infected HCC patients. (**E**) Inhibition of hepatitis B virus replication could up‐regulate the expression of miR‐384 in HBV‐related HCC. (**F**) The correlation between miR‐384 serum levels and PTN serum levels in HCC patients and healthy persons. A significant negative correlation was observed between miR‐384 serum levels and PTN serum levels (*r* = −0.6435, *P* < 0.0001)

We measured serum PTN levels in 15 healthy volunteers, 25 patients with HBV‐related hepatitis, 17 patients with HBV‐related cirrhosis, 20 patients with HBV‐related HCC and 11 patients with non‐HBV‐related HCC (Fig. 4A). The median serum PTN level in the 15 healthy volunteers was 267.65 pg/ml (mean = 267.65 ± 107.20 pg/ml). The median serum PTN level in the 25 patients with HBV‐related hepatitis was 981.29 pg/ml (mean = 981.29 ± 261.82 pg/ml). The median serum PTN level in the 17 patients with HBV‐related cirrhosis was 1294.20 pg/ml (mean = 1294.20 ± 180.57 pg/ml). The median serum PTN level in the 20 patients with HBV‐related HCC was 1173.66 pg/ml (mean = 1173.66 ± 209.57 pg/ml). The median serum PTN level in the 11 patients with non‐HBV‐related HCC was 1008.52 pg/ml (mean = 1008.52 ± 205.17 pg/ml). The median serum PTN level in the 20 patients with HBV‐related HCC who were treated with entecavir was 1036.65 pg/ml (mean = 1036.65 ± 174.21 pg/ml). These results showed that PTN expression was higher in HCC tissues than in healthy volunteers, and it gradually increased with the transition from hepatitis to cirrhosis (Fig. 4A) and slightly declined from liver cirrhosis to HCC. The serum PTN level was highest in patients with HBV‐related cirrhosis (Fig. 4A). The results further suggest that PTN plays an important role in the evolutionary processes from hepatitis, liver cirrhosis to HCC caused by HBV, particularly the transition from liver cirrhosis to HCC. Inhibition of HBV replication could down‐regulate the expression of PTN (Fig. 4B). This finding suggests that HBV may affect the expression level of PTN. However, the specific mechanism remains unknown.

Blood samples were collected pre‐operatively and post‐operatively at 2, 14and 28 days from 17 HCC patients treated with TACE. The results showed that the serum PTN levels were slightly elevated at 2 days post‐operatively in the blood compared with the pre‐operative blood samples (*P* > 0.05), but a gradual decline was observed in the 14 and 28‐day post‐operative blood samples. The study found that reduced serum PTN levels were closely related to the treatment effect of TACE. A subsequent rise in PTN serum levels would suggest residual tumour recurrence (Fig. 4C).

We also detected miR‐384 serum levels in the 15 healthy volunteers, 25 HBV‐related hepatitis patients, 17 HBV‐related cirrhosis patients, 20 HBV‐related HCC patients and 11 non‐HBV‐infected HCC patients (Fig. 4D). Serum miR‐384 levels were highest in the healthy volunteers and lowest in the HBV‐related HCC patients. miR‐384 serum levels were up‐regulated in response to the inhibition of HBV replication (Fig. 4E).

The correlation between miR‐384 serum levels and PTN serum levels in HCC patients and healthy persons revealed a significant negative correlation between miR‐384 and PTN (*r* = −0.6435, *P* < 0.0001; Fig. 4F).

**Figure 5:**
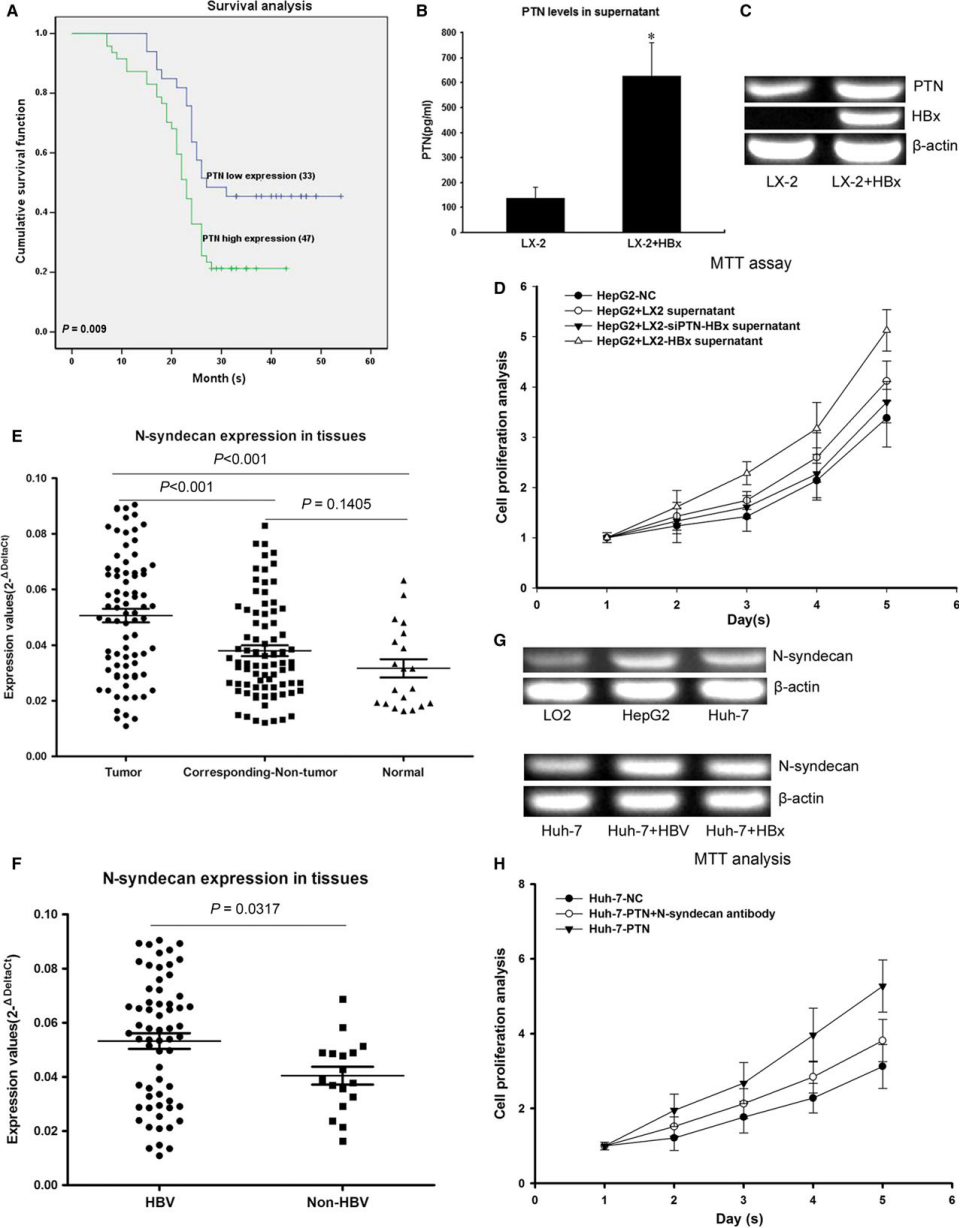
HBx stimulates the growth of hepatocytes through PTN expression, and the PTN receptor N‐syndecan is highly expressed in HCC tissues. (**A**) Kaplan–Meier analysis showing that HCC patients with high PTN expression presented significantly shorter survival time than those with low PTN expression. (**B**) Comparative analysis of PTN levels in the supernatant of LX‐2‐HBx and control cells by ELISA. PTN levels were higher in LX‐2‐HBx than in controls**.** (**C**) RT‐PCR results showing that PTN mRNA levels were higher in LX‐2‐HBx than in controls**.** (**D**) HepG2 displayed the most rapid proliferation in the supernatant of LX‐2‐HBx. The expression of PTN was inhibited by RNAi in LX‐2‐HBx cells and cocultured with HepG2. This result showed that the proliferation of HepG2 cocultured with LX‐2‐HBx‐siPTN was reduced compared with the control. (**E**) Analysis of 80 paired tumour tissue samples, adjacent non‐tumour tissue samples and 20 normal tissue samples showed that the expression of N‐syndecan was increased in tumour tissues compared with non‐tumour and normal tissues. (**F**) HBV‐infected HCC patients showed higher expression levels of N‐syndecan compared with that in HBV‐negative HCC patients. (**G**) The expression of N‐syndecan in HepG2 and Huh‐7 was higher than that in LO2 normal hepatocytes, and the expression of N‐syndecan was up‐regulated in HBV‐infected cells and HBx‐transfected cells compared with control cells. (**H**) Antibody inhibition of N‐syndecan could impede hepatocyte proliferation induced by PTN. *P < 0.05

Kaplan–Meier analysis showed that HCC patients with high PTN expression levels presented a significantly shorter survival time than those with low PTN expression (χ^2^ **=** 6.887, *P* = 0.009; Fig. 5A, *P* < 0.05).

### LX‐2 transfected with HBx stimulates hepatocyte growth through PTN expression

LX‐2 cells were transfected with HBx and cocultured with HepG2, and the proliferation of HepG2 cells was detected by MTT. Comparative analysis of PTN levels in the supernatant of LX‐2‐HBx and control cells by ELISA revealed higher PTN levels in LX‐2‐HBx compared with control cells (Fig. 5B and C). PTN enhanced the proliferation of HepG2 in coculture (Fig. 5D).

### The PTN receptor N‐syndecan is highly expressed in HCC tissues

*N‐*syndecan, PTPζ and ALK are known as PTN receptors, regardless of whether their expression is correlated to PTN. We found that N‐syndecan was highly expressed in HCC tissues (Fig. 5E). HBV‐infected HCC patients showed higher expression levels of N‐syndecan than in HBV‐negative HCC patients (Fig. 5F). The expression of N‐syndecan in HepG2 and Huh‐7 was higher than that in LO2 normal hepatocytes, and the expression of N‐syndecan was up‐regulated in HBV‐infected cells and HBx‐transfected cells compared with control cells (Fig. 5G). Furthermore, antibody‐induced inhibition of N‐syndecan could impede Huh‐7 proliferation induced by PTN (Fig. 5H). This correlation supports the conclusion that N‐syndecan functions as a PTN receptor in hepatocytes. Considering these results together, PTN acted as a growth factor *via* N‐syndecan on hepatocytes, further promoting cell proliferation, metastasis and lipogenesis.

### MiR‐384 and PTN affect cell proliferation, colony formation, invasion and metastasis *in vitro*

**Figure 6:**
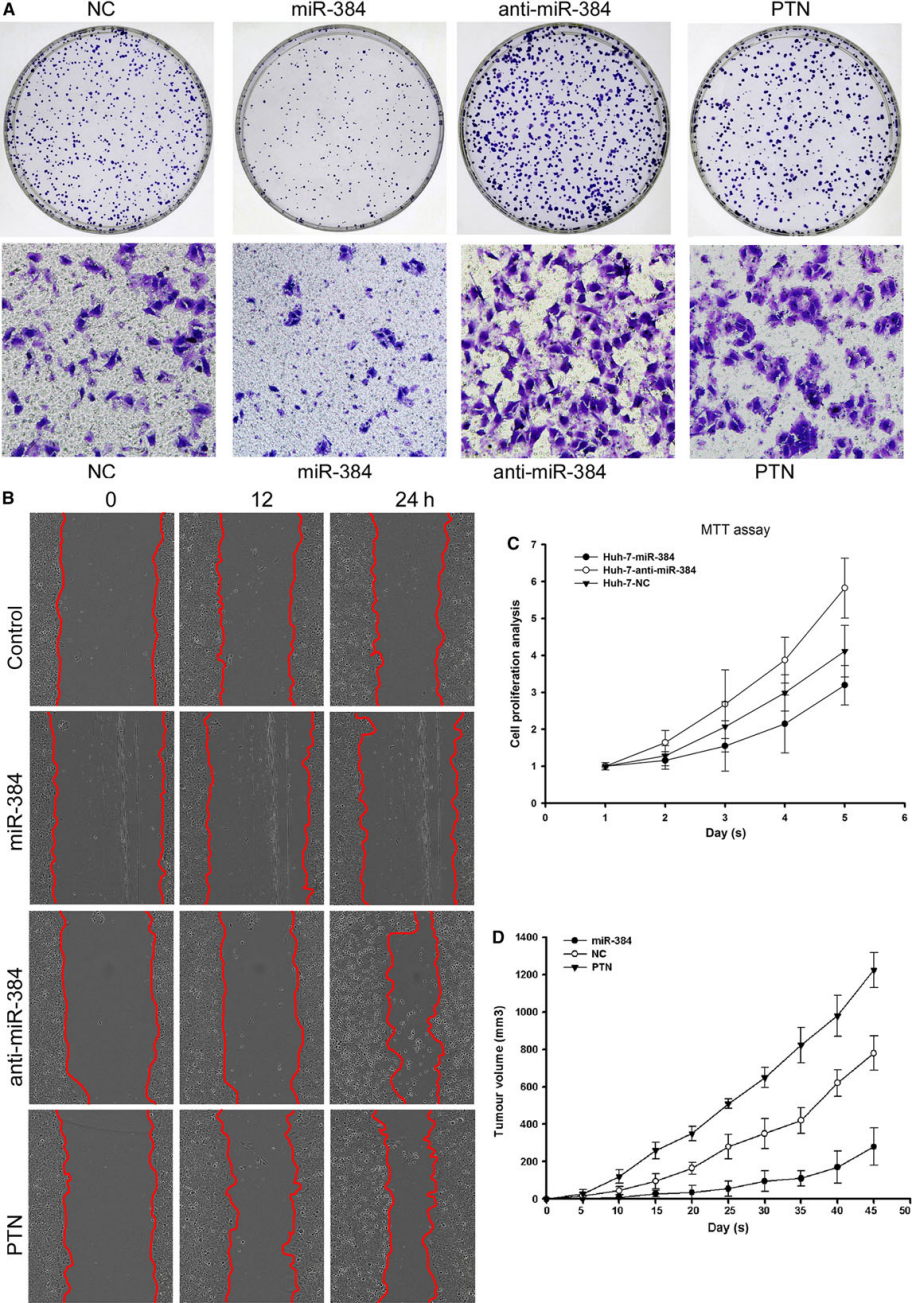
MiR‐384 and PTN affect cell proliferation, colony formation, invasion and metastasis *in vitro*, and miR‐384 and PTN affect tumour growth *in* vivo. (**A**) miR‐384 could inhibit the colony formation and invasion ability of Huh‐7 compared with control cells. Anti‐miR‐384 and PTN increased the colony formation and invasion ability of Huh‐7 cells compared with the controls. (**B**) miR‐384 could inhibit the metastasis ability of Huh‐7 cells compared with the controls. Anti‐miR‐384 and PTN increased the metastasis ability of Huh‐7 cells compared with the controls. (**C**) The cell growth curve clearly showed decreased proliferation in Huh‐7‐ miR‐384 compared with Huh‐7‐NC cells. Huh‐7‐anti‐miR‐384 displayed increased proliferation compared with Huh‐7‐NC cells. (**D**) The tumour volume in the PTN overexpression group was significantly greater than that in the control group. The tumour nodule volume in the miR‐384 overexpression group was significantly smaller than that in the control group.

The roles of miR‐384 and PTN in the formation of cell colonies were investigated by soft‐agar colony formation assays. We found that miR‐384 could inhibit the colony formation ability of Huh‐7 cells compared with the controls. Anti‐miR‐384 and PTN increased the colony formation ability of Huh‐7 cells (Fig. 6A).

Transwell assays were performed to explore the effects of miR‐384 and PTN on HCC cell invasion. The results indicated that overexpression of miR‐384 could inhibit the invasion of Huh‐7 cells and that anti‐miR‐384 and PTN could promote the invasion potential of Huh‐7 cells *in vitro* (Fig. 6A).

For the wound‐healing assays, miR‐384 could inhibit the metastasis ability of Huh‐7 cells compared with the controls. Anti‐miR‐384 and PTN increased the metastasis ability of Huh‐7 cells compared with the controls (Fig. 6B).

MTT assays were performed to assess the role of miR‐384 in HCC cell proliferation. The cell growth curve clearly showed decreased proliferation in Huh‐7‐miR‐384 compared with Huh‐7‐NC cells. Huh‐7‐anti‐miR‐384 showed increased proliferation compared with Huh‐7‐NC cells (Fig. 6C).

### MiR‐384 and PTN affect tumour growth and angiogenesis in nude mice

**Figure 7:**
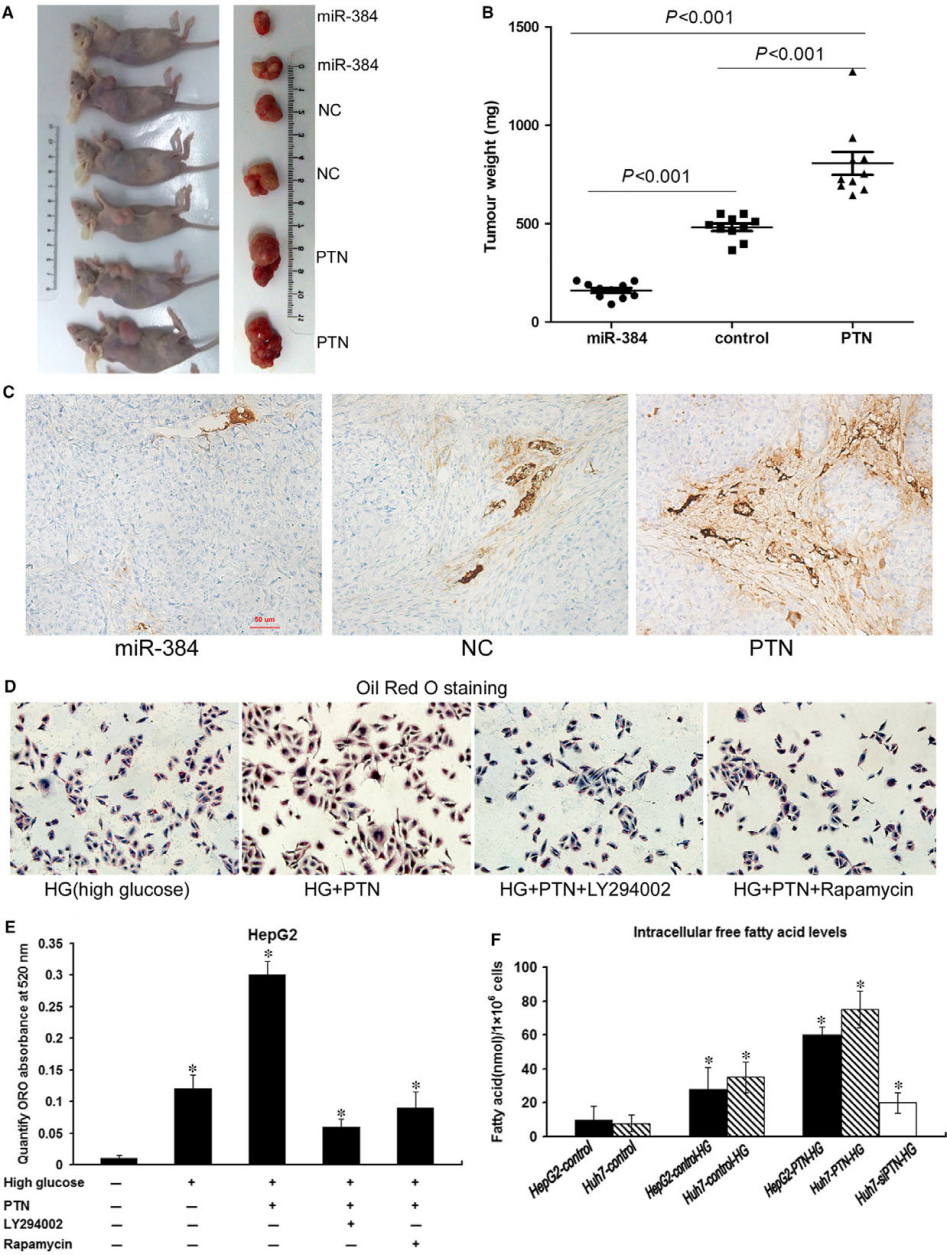
MiR‐384 and PTN affect tumour growth and angiogenesis *in* vivo, and PTN promotes lipogenesis. (**A**) Huh‐7 cells (5 × 10^6^) transfected with miR‐384, PTN and controls were implanted subcutaneously into the flank of nude mice. The mice were killed 45 days later, and tumour nodules were removed and photographed. (**B**) The tumour nodule weight in the PTN overexpression group was greater than that in the control group. The tumour nodule weight in the miR‐384 overexpression group was smaller than that in the control group. (**C**) Immunohistochemical staining with CD34 antibody was conducted to analyse the microvessel density in tumour nodules. The density of tumour blood vessels observed in PTN overexpression tumour nodules greatly exceeded that in the control group and the miR‐384 overexpression group. (**D**) The cells were stained with Oil Red O. Lipogenesis induced by high glucose was much higher in HepG2‐PTN than in HepG2‐NC. The lipogenesis induced by high glucose in HepG2‐PTN was inhibited by LY294002 and rapamycin**.** (**E**) ORO staining was quantified by measuring the absorbance at 520 nm. The lipid content of HepG2‐PTN cells was significantly higher than that of the parental control cells, and LY294002 and rapamycin could diminish lipogenesis. (**F**) The high sugar could promote free fatty acid synthesis. The intracellular free fatty acid levels in PTN‐transfected cells were higher than those in control cells. Down‐regulation of PTN expression weakened HG‐induced lipogenesis. *P < 0.05

To confirm the *in vitro* phenotype of miR‐384 and PTN, we examined the effect of miR‐384 and PTN in a nude mouse tumour model. Consistent with the results obtained *in vitro*, the tumour volume and tumour weight in the PTN overexpression group were significantly greater than those in the control group (Figs 6D, 7A and B). However, the tumour volume and tumour weight in the miR‐384 overexpression group were less than those in the control group (Figs 6D, 7A and B).

Immunohistochemical staining with CD34 antibody was conducted to analyse the microvessel density in tumour nodules of miR‐384, PTN and control groups, and these microvessels (at 20×) were counted in five random microscopic fields to assess the tumour nodules in every group. The mean ± S.D. microvessel density was 3.35 ± 1.77 in tissues expressing miR‐384, 5.64 ± 2.14 in tissues in the control group and 11.69 ± 3.38 in tissues expressing PTN. The microvessel density was significantly increased in tumour nodules expressing high levels of PTN compared with miR‐384 and control group tumour nodules (*P* < 0.05; Fig. 7C) (Table 3).

**Table 3:** The immunohistochemistry stained with CD34 antibody to analysis and count these microvessels (at 20 × ) in five random microscopic field for tumour nodules of miR‐384, PTN and control groups

Pathological factors	*N*	Microvessel density	χ^2^
miR‐384	10	3.35 ± 1.77	*P* < 0.05^a^
Control	10	5.64 ± 2.14	
PTN	10	11.69 ± 3.38	

a *P* < 0.05 was considered statistically significant.

### PTN promotes hepatoma cell lipogenesis

Analysis of the immunohistochemical results revealed that the expression of PTN was increased in some patients with steatosis (Fig. 3A). This finding revealed whether PTN was related to abnormal lipid metabolism in hepatoma cells. We aimed to determine the role of PTN in hepatoma cell lipogenesis. Because a high sugar content could promote cell lipogenesis, the cells that were transfected with PTN induced in 25 mM glucose (high glucose; HG) were stained with Oil Red O, and the results showed that lipogenesis induced by HG was much higher in HepG2‐PTN than in HepG2‐NC. The lipogenesis induced by HG in HepG2‐PTN was inhibited by LY294002 and rapamycin (Fig. 7D and E). The intracellular free FA levels in PTN‐transfected cells were higher than those in control cells, Down‐regulation of PTN expression weakened HG‐induced lipogenesis (Fig. 7F).

### PTN promotes proliferation, metastasis and lipogenesis through activation of the PI3K/AKT/mTORC1 pathway

**Figure 8:**
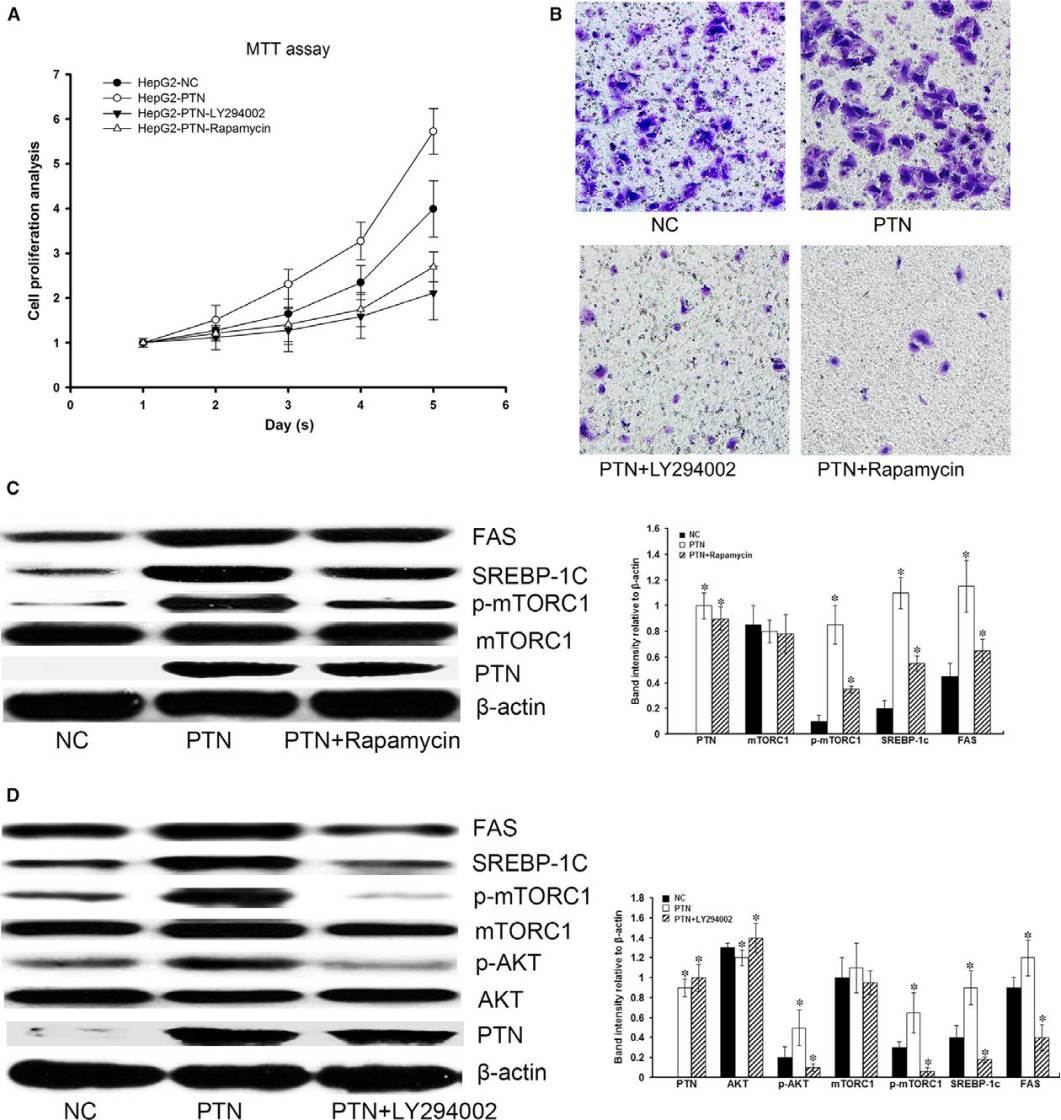
PTN promotes proliferation, metastasis and lipogenesis through activation of the PI3K/AKT/mTORC1 pathway. (**A and B**) PTN could promote cell proliferation and metastasis, but the proliferation and metastasis induced by PTN were inhibited by LY294002 and rapamycin**.** (**C**) PTN could increase the level of mTORC1 phosphorylation. PTN through mTORC1 increased the expression of SREBP‐1c and FAS in HepG2‐PTN cells compared with control cells. However, the expression of SREBP‐1c and FAS induced by PTN was inhibited by rapamycin. (**D**) PTN could increase the level of AKT phosphorylation. LY294002 could inhibit the level of AKT and mTORC1 phosphorylation. The expression of SREBP‐1c and FAS induced by PTN was inhibited by LY294002. Our results indicated that PTN regulated SREBP‐1c and FAS expression through the PI3K/Akt/mTORC1 pathway to promote lipogenesis. *P < 0.05

To identify which of the many signalling pathways downstream of PTN regulates lipogenesis, HepG2‐PTN cells were treated with 10 μm LY294002 (PI3K/AKT inhibitor) or 50 nm rapamycin (mTORC1 inhibitor). The proliferation and metastasis induced by PTN were inhibited by LY294002 and rapamycin (Fig. 8A and B). PTN increased the expression of SREBP‐1c in HepG2‐PTN cells compared with control cells. The expression level of FAS, which was identified as the target gene of SREBP‐1c, was also significantly increased in PTN‐transfected hepatoma cells compared with control cells (Fig. 8C). PTN also increased the phosphorylation of AKT and mTORC1 in HepG2‐PTN cells compared with control cells (Fig. 8D). However, the expression levels of SREBP‐1c and FAS induced by PTN were inhibited by LY294002 and rapamycin (Fig. 8C and D). These results suggested that PTN could up‐regulate the expression of SREBP‐1c and FAS through activation of the PI3K/AKT/mTORC1 pathway.

### High glucose may promote lipogenesis in hepatocytes through the AP‐1/PTN/PI3K/Akt/mTORC1 pathway. miR‐384 could inhibit high glucose‐induced lipogenesis in hepatocytes

**Figure 9:**
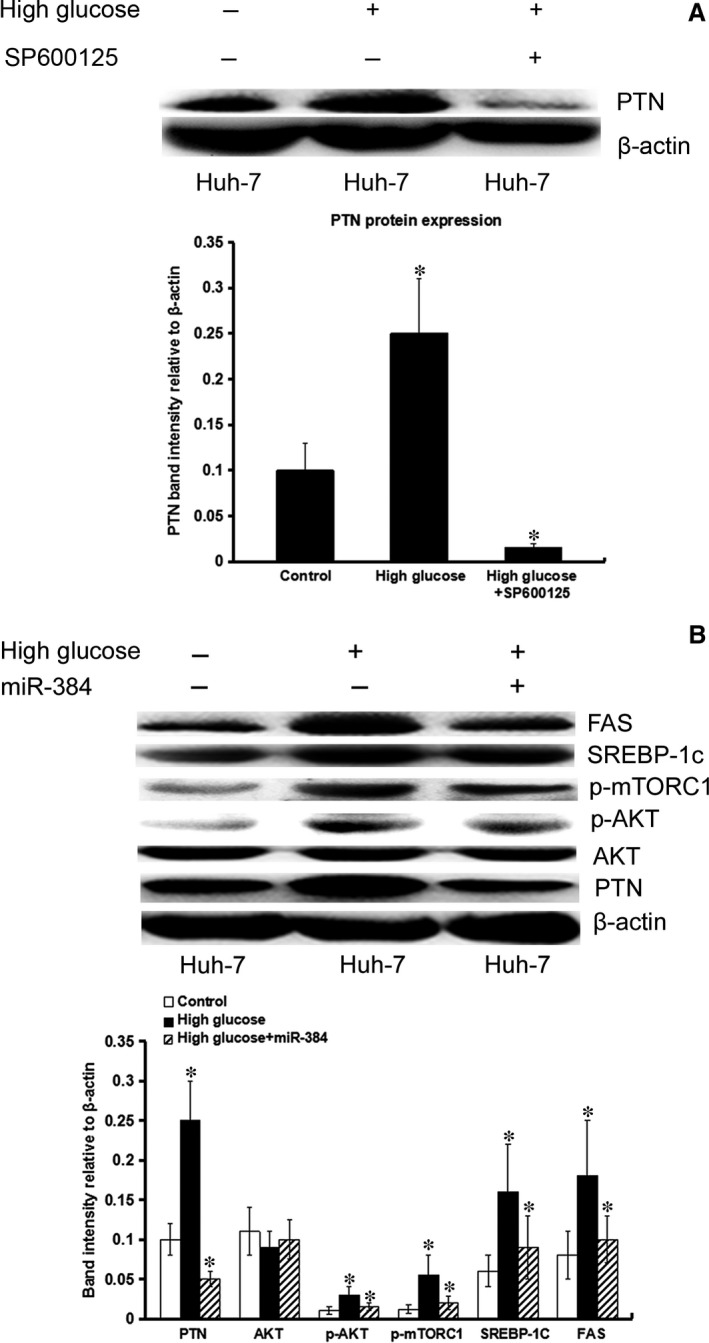
High glucose may promote the expression of PTN and lipogenesis of hepatocyte. miR‐384 plays an important role in the lipogenesis of hepatocytes. (**A**) High glucose may promote PTN expression. SP600125 suppresses high glucose‐induced PTN expression. (**B**) High glucose triggered Akt phosphorylation and activated downstream mTORC1, leading to increased triglyceride levels and up‐regulation of the expression of lipogenic genes such as SREBP‐1c, FAS. Using high glucose‐treated hepatocytes as control cells, our results showed that the expression of PTN, SREBP1c and FAS, as well as the levels of AKT and mTORC1 phosphorylation, was decreased in high glucose‐induced Huh‐7 cells treated with miR‐384 compared with control cells. *P < 0.05.

Our results showed that HG‐induced activation of the AP‐1 pathway could increase the expression of PTN in hepatocytes. The AP‐1 inhibitor, SP600125, suppressed HG‐induced PTN expression by inhibiting the AP‐1‐mediated pathway (Fig. 9A). This finding supported an important role of the AP‐1/PTN pathway in the regulation of *de novo* lipogenesis induced by HG. HG triggered Akt phosphorylation and activated downstream mTORC1, leading to increased triglyceride levels and an up‐regulation of the expression of lipogenic genes such as SREBP1c and FAS (Fig. 9B). Therefore, we used HG‐treated hepatocytes as control cells. Our results showed that the expression of PTN, SREBP1c and FAS, and the levels of AKT and mTORC1 phosphorylation, was decreased in HG‐induced hepatocytes treated with miR‐384 compared with control cells. This result verified that miR‐384, as an upstream molecule of PTN with the ability to influence lipid metabolic pathways, functions to inhibit the synthesis of lipids (Fig. 9B).

## Discussion

HBV infection has been identified as a risk factor for HCC. The available experimental evidence suggests that HBV has multifunctional activity that plays a key role in the regulation of liver cell proliferation, metastasis and lipid metabolic abnormalities 5, 11, 12. Although the exact molecular mechanisms are not clear, numerous studies have suggested that HBx plays a pivotal role in HBV‐induced liver pathogenesis by altering the expression of miRNAs that are associated with hepatic steatosis, fibrosis and malignant transformation 13, 14. The dysregulation of miRNA expression is also associated with abnormal FAS and metabolism 15, 16. However, the roles of miRNAs in HCC proliferation, metastasis and lipid metabolic abnormalities remain largely unknown. In this study, we observed a down‐regulation of miR‐384 expression in HCC tissues compared with non‐tumourous tissues.

However, research investigating the functions of miR‐384 in tumours is very scarce, necessitating further investigations. Ding and colleagues showed that miR‐384 was down‐regulated in human colorectal cancer 17. Xu and colleagues showed that miR‐384 was significantly down‐regulated in HCC. Their studies showed that insulin receptor substrate 1 (IRS1) was identified as a direct and functional target of miR‐384. miR‐384 decreased IRS1 expression, subsequently down‐regulating cyclin D1 and up‐regulating p21 and p‐Rb expression to suppress cellular proliferation in HCC 18. In the present study, HBV‐infected HCC patients showed lower expression levels of miR‐384 compared with HBV‐negative HCC patients. The expression of miR‐384 was negatively correlated with HBV infection. Further studies showed that the decreased level of miR‐384 was significantly correlated with HBV infection, adjacent organ invasion, microscopic vascular invasion and advanced TNM tumour stage. The low expression of miR‐384 was correlated with poor clinical features of HCC patients. The data from this study suggested that miR‐384 acted as a tumour suppressor in HCC and that the down‐regulation of miR‐384 contributed to metastasis and tumour progression in HCC patients.

To further understand the possible function of miR‐384 in HBV‐related HCC, we identified PTN as a potential miR‐384 target gene. There was an inverse correlation between PTN and miR‐384 expression in HCC patients. Overexpression of PTN increased cellular proliferation and metastasis. Overexpression of PTN abrogated miR‐384 induced cell growth and invasion inhibition. As an oncogene, PTN is overexpressed in a number of human cancers, and its overexpression contributes to malignant transformation by regulating the expression of a number of genes that participate in multiple aspects of tumorigenesis, such as angiogenesis, cell cycle progression, cell invasion, migration, metastasis and angiogenesis 19, 20, 21, 22. PTN is also a strong mitogen of hepatocytes and is involved in liver regeneration 23, 24, 25. The IHC results showed that PTN was expressed in HSCs, hepatocytes and hepatoma cells. PTN expression was up‐regulated in HCC tissues compared with adjacent non‐tumour tissues, and HBx could regulate PTN expression by inhibiting miR‐384. The statistical analysis showed that increased expression of PTN was correlated with HBV infection, cirrhosis, adjacent organ invasion, microscopic vascular invasion and advanced TNM stage. Our results suggest that PTN plays an important role in the evolutionary processes from hepatitis, liver cirrhosis to HCC caused by HBV, particularly in the transition from liver cirrhosis to HCC.

In the present study, we found a negative correlation between PTN and miR‐384. Because PTN and miR‐384 can be detected in the serum, they may be used in clinical applications as indicators in hepatocarcinoma screening, relapse and prognosis. However, there are overlaps in serum levels of PTN and miR‐384 between normal persons and tumour patients. When the two indicators are applied, false‐positive and false‐negative cases may be encountered. However, a comprehensive analysis of PTN and miR‐384 may be a better indicator in hepatocarcinoma screening, relapse and prognosis.

In HSC cultures, PTN transcription was significantly increased in response to HBx, and PTN enhanced the growth of hepatocytes in coculture. This finding suggested that HSCs in HBV infection stimulate the growth of hepatocytes through PTN expression. Each N‐syndecan, RPTPβ/ζ and ALK has been reported to function as a PTN receptor, regardless of whether the expression is correlated with that PTN 20, 21, 26. Asahina and colleagues 27 showed transient but marked increased expression of N‐syndecan during the initial phase of D‐galactosamine (GalN) treatment. This stimulation pattern correlated well with that of PTN mRNA. However, it should be noted that the expression of RPTPβ/ζ was extremely low compared with that of N‐syndecan in the liver. ALK mRNA was not detectable in any of these liver regeneration models. Therefore, Yoshizato 27 assumed that ALK does not act as the PTN receptor, at least in the liver. However, the present findings demonstrated that hepatocytes in culture spontaneously largely increase the expression of N‐syndecan mRNA. Thus, it is suggested that PTN/N‐syndecan signalling functions in cultured hepatocytes and stimulates their growth. This correlation supports the function of N‐syndecan as a PTN receptor in hepatocytes. Based on the experimental results of Yoshizato 27, as a PTN receptor, N‐syndecan plays an important role in liver regeneration. We speculate that N‐syndecan may play the same important role in HCC.

N‐syndecan is a cell surface heparan sulphate proteoglycan. Membrane‐bound heparan sulphate proteoglycans act as coreceptors for cytokines and are involved in proliferation or cell–cell adhesion 28. Previous studies have found that N‐syndecan levels are increased in bladder cancer tissues 29. The high expression level of PTN combined with N‐syndecan may contribute to the increased perineural invasion and poor prognosis of pancreatic cancer 30. N‐syndecan is also known to regulate the energy balance. In particular, N‐syndecan has been implicated in the modulation of lipogenesis and promotion of FAS 28, 31. We observed high expression levels of N‐syndecan in HCC. The expression of N‐syndecan was positively correlated with HBV infection. HBV could up‐regulate N‐syndecan expression, suggesting that the high expression level of N‐syndecan in HBV‐related HCC may be related to carcinogenesis and hepatocellular steatosis induced by HBV. Additionally, inhibition of N‐syndecan could impede hepatocyte proliferation induced by PTN. Considering these results together, PTN acts as a growth factor *via* N‐syndecan on hepatocytes to promote cell proliferation, metastasis and lipogenesis.

We focused our study on the effect of miR‐384 and PTN for the modulation of hepatoma cell proliferation and metastasis. The up‐regulation of PTN expression could promote hepatoma cell proliferation and metastasis. In addition, an enhanced tumour volume and density of tumour blood vessels were observed in PTN‐transfected tumour models relative to controls *in vivo*. The activation of N‐syndecan/PI3K/AKT/mTORC1 pathways was regulated by PTN in HCC, which was considered the key mechanism underlying the hepatoma cell proliferative and migratory capacity induced by HBx.

The immunohistochemical results showed that the expression of PTN was increased in some patients with steatosis, suggesting that PTN was related to abnormal lipid metabolism in hepatoma cells. We found that HBx‐induced lipogenesis and expression of SREBP‐1c were largely reduced when the expression of PTN was repressed by RNA interference. The expression of FAS, the target gene of SREBP‐1c, was also decreased; however, the effect of this PTN on the expression of SREBP‐1c requires further study. The results showed that PTN could increase the phosphorylation of AKT and mTORC1, and the expression of SREBP‐1c and FAS induced by PTN was down‐regulated by LY294002 and rapamycin. This finding suggested that, through the N‐syndecan/PI3K/Akt/mTORC1 pathway, PTN could promote the expression of the SREBP‐1c gene, further facilitating *de novo* lipogenesis by up‐regulating the lipogenic enzyme FAS. Up‐regulation of FAS, the key metabolic multi‐enzyme responsible for the terminal catalytic step in FAS 32, represents a phenotypic alteration in many human malignancies including HCC 33.

Recent evidence 34, 35, 36 has shown that treatment with 25 mM glucose HG increases cellular *de novo* lipogenesis compared with normal 5.5 mM glucose. HG triggered Akt phosphorylation and activated downstream mTORC1, leading to increased triglyceride levels and up‐regulation of the expression of lipogenic genes such as SREBP1c, FAS and ACC. HG treatment also stimulated lipid accumulation and the triglyceride contents in hepatocytes. In addition, HG could induce the phosphorylation of Akt and mTORC1 in a time‐dependent manner. Pre‐treatment with LY294002 and rapamycin blocked HG‐induced *de novo* lipogenesis and lipogenic gene expression. The above results revealed that HG could activate the PI3K/Akt/mTOR pathway to mediate HG‐induced lipogenesis in hepatocytes.

Some evidence 37, 38 has demonstrated HG‐induced transcriptional activity of activator protein‐1 (AP‐1). In the distal 5′‐region of the PTN promoter, two binding sites for the transcription factor AP‐1 were found 39, 40, 41, 42, and the two AP‐1 binding sites of the PTN promoter are involved in the HG‐induced stimulation of its expression. Our result showed that HG‐induced activation of the AP‐1 pathway can increase the expression of PTN in hepatocytes. The AP‐1 inhibitor, SP600125, suppresses HG‐induced PTN expression by inhibiting the AP‐1‐mediated pathway, which suggested an important role for the AP‐1/PTN pathways in the regulation of *de novo* lipogenesis induced by HG.

Some researches 43, 44, 45, 46 have shown that chronic viral infections, such as HBV, may decrease the tissue response to insulin, thereby causing insulin resistance. This phenomenon leads to abnormal blood glucose metabolism in liver tissue, suggesting that hyperglycaemia may promote the lipogenesis of hepatocytes through the AP‐1/PTN/N‐syndecan/PI3K/Akt/mTORC1 pathway.

In general, human cancer cells exhibit high levels of lipogenesis because lipogenesis is essential for them to obtain sufficient lipids for energy production and membrane biogenesis; accelerated FAS is important for cellular proliferation and metastasis 47, 48, 49, 50. The above results showed that PTN was an important lipid regulatory gene and promoted the synthesis of FAs.

However, Gu and Yi 51, 52 showed that PTN could inhibit pre‐adipocyte 3T3‐L1 differentiation *via* the β‐catenin pathway, which is inconsistent with our results. The reason for the opposite results for hepatoma cells compared with pre‐adipocytes regulated by PTN in lipid metabolism remains unknown, but many studies have detected differences in energy metabolism between tumour cells and normal cells. However, specific factors are not clear. Together with published results, mutations and abnormal activity of β‐catenin are most commonly observed in HCC 53, 54. Wnt/β‐catenin plays an important role in mediating the repression of pre‐adipocyte differentiation 55, 56. We hypothesized that the differing roles of PTN in lipid metabolism resulted from differences in β‐catenin between tumour cells and normal cells. It has been suggested that other lipid regulatory pathways may be more important in hepatoma cells. Our results revealed that the HBx/miR‐384/PTN/N‐syndecan/PI3K/Akt/mTORC1/SREBP‐1c/FAS pathway plays an important role in HBx‐induced hepatic lipogenesis. However, further experiments might be necessary to identify the molecular mechanisms underlying the dysregulation of PTN in lipid metabolism in tumour cells and normal cells.

In conclusion, this study confirmed that the loss of miR‐384 was a common event in HCC, especially in HBV‐related HCC. We further provided evidence for a role of miR‐384, a microRNA with potential tumour suppressor activity, in the negative regulation of the expression of PTN, an important trophic factor in cell proliferation that is considered to facilitate the progression of HCC and promote the metastasis of cells. Our results showed that PTN functions through the PI3K/Akt/mTORC1 pathway to regulate SREBP‐1c and FAS expression to promote lipogenesis. Another study showed that the PTN receptor N‐syndecan is highly expressed in HCC. The expression of N‐syndecan was positively correlated with HBV infection. HBV could up‐regulate N‐syndecan expression. PTN acts as a growth factor *via* N‐syndecan on hepatocytes to promote cellular proliferation, metastasis and lipogenesis. Therefore, our study provides important information that improves our understanding of the mechanisms underlying HBV‐mediated steatosis, cirrhosis and the development of HCC. PTN may thus represent a new potential therapeutic target for the prevention of hepatic steatosis and further progression to HCC after chronic HBV infection.

## Supporting information


**Figure S1** The inhibition of exosome secretion through GW4869 could reduce the PTN expression in LX‐2 co‐cultured with HepG2‐HBx.

## References

[1] Wang MD , Wu H , Huang S , *et al* HBx regulates fatty acid oxidation to promote hepatocellular carcinoma survival during metabolic stress. Oncotarget. 2016; 7: 6711–26. 26744319 10.18632/oncotarget.6817 PMC4872744

[2] Liao X , Han C , Qin W , *et al* Genome‐wide association study identified PLCE1‐ rs2797992 and EGFR‐ rs6950826 were associated with TP53 expression in the HBV‐related hepatocellular carcinoma of Chinese patients in Guangxi.Am. J Transl Res. 2016; 8: 1799–812. PMC4859909 27186304

[3] Na TY , Shin YK , Roh KJ , *et al* Liver X receptor mediates hepatitis B virus X protein‐induced lipogenesis in hepatitis B virus‐associated hepatocellular carcinoma. Hepatology. 2009; 49: 1122–31. 19105208 10.1002/hep.22740

[4] Chao CC . Inhibition of apoptosis by oncogenic hepatitis B virus X protein: implications for the treatment of hepatocellular carcinoma. World J Hepatol. 2016; 8: 1061–6. 27660672 10.4254/wjh.v8.i25.1061 PMC5026997

[5] You X , Liu F , Zhang T , *et al* Hepatitis B virus X protein upregulates oncogene Rab18 to result in the dysregulation of lipogenesis and proliferation of hepatoma cells. Carcinogenesis. 2013; 34: 1644–52. 23471881 10.1093/carcin/bgt089

[6] Peck B , Schulze A . Lipid desaturation ‐ the next step in targeting lipogenesis in cancer? FEBS J. 2016; 283: 2767–78. 26881388 10.1111/febs.13681

[7] Mounier C , Bouraoui L , Rassart E . Lipogenesis in cancer progression. Int J Oncol. 2014; 45: 485–92. 24827738 10.3892/ijo.2014.2441

[8] Wang L , Yue Y , Wang X , *et al* Function and clinical potential of microRNAs in hepatocellular carcinoma. Oncol Lett. 2015; 10: 3345–53. 26788134 10.3892/ol.2015.3759 PMC4665873

[9] Li CH , Chow SC , Yin DL , *et al* Functional characterisation of hepatitis B viral X protein/microRNA‐21 interaction in HBV‐associated hepatocellular carcinoma. Hong Kong Med J. 2016; 22: 37–42. 27390009

[10] Li J , Huang Q , Long X , *et al* CD147 reprograms fatty acid metabolism in hepatocellular carcinoma cells through Akt/mTOR/SREBP1c and P38/PPARα pathways. J Hepatol. 2015; 63: 1378–89. 26282231 10.1016/j.jhep.2015.07.039

[11] Huang Y , Wang Z , An S , *et al* Role of hepatitis B virus genotypes and quantitative HBV DNA in metastasis and recurrence of hepatocellular carcinoma. J Med Virol. 2008; 80: 591–7. 18297705 10.1002/jmv.21117

[12] Teng CF , Hsieh WC , Yang CW , *et al* A biphasic response pattern of lipid metabolomics in the stage progression of hepatitis B virus X tumorigenesis. Mol Carcinog. 2016; 55: 105–14. 25594851 10.1002/mc.22266

[13] Zhang D , Wang Y , Ji Z , *et al* Identification and differential expression of microRNAs associated with fat deposition in the liver of Wistar rats with nonalcoholic fatty liver disease. Gene. 2016; 585: 1–8. 26971813 10.1016/j.gene.2016.03.011

[14] Chen WS , Yen CJ , Chen YJ , *et al* miRNA‐7/21/107 contribute to HBx‐induced hepatocellular carcinoma progression through suppression of maspin. Oncotarget. 2015; 6: 25962–74. 26296971 10.18632/oncotarget.4504 PMC4694878

[15] Man XF , Tan SW , Tang HN , *et al* MiR‐503 inhibits adipogenesis by targeting bone morphogenetic protein receptor 1a. Am J Transl Res. 2016; 8: 2727–37. 27398155 PMC4931166

[16] Ahn J , Lee H , Chung CH , *et al* High fat diet induced downregulation of microRNA‐467b increased lipoprotein lipase in hepatic steatosis. Biochem Biophys Res Commun. 2011; 414: 664–9. 21986524 10.1016/j.bbrc.2011.09.120

[17] Wang YX , Chen YR , Liu SS , *et al* MiR‐384 inhibits human colorectal cancer metastasis by targeting KRAS and CDC42. Oncotarget. 2016; 7: 84826–38. 27769041 10.18632/oncotarget.12704 PMC5356701

[18] Lai YY , Shen F , Cai WS , *et al* MiR‐384 regulated IRS1 expression and suppressed cell proliferation of human hepatocellular carcinoma. Tumor Biol. 2016; 37: 14165–71. 10.1007/s13277-016-5233-5 27542674

[19] Ball M , Carmody M , Wynne F , *et al* Expression of pleiotrophin and its receptors in human placenta suggests roles in trophoblast life cycle and angiogenesis. Placenta. 2009; 30: 649–53. 19481257 10.1016/j.placenta.2009.05.001

[20] Maeda N , Noda M . Involvement of receptor‐like protein tyrosine phosphatase zeta/RPTP beta and its ligand pleiotrophin/heparin‐binding growth‐associated molecule (HB‐GAM) in neuronal migration. J Cell Biol. 1998; 142: 203–16. 9660874 10.1083/jcb.142.1.203 PMC2133018

[21] Koyama‐Nasu R , Haruta R , Nasu‐Nishimura Y , *et al* The pleiotrophin‐ALK axis is required for tumorigenicity of glioblastoma stem cells. Oncogene. 2014; 33: 2236–44. 23686309 10.1038/onc.2013.168

[22] Kong Y , Bai PS , Nan KJ , *et al* Pleiotrophin is a potential colorectal cancer prognostic factor that promotes VEGF expression and induces angiogenesis in colorectal cancer. Int J Colorectal Dis. 2012; 27: 287–98. 22065111 10.1007/s00384-011-1344-z

[23] Michelotti GA , Tucker A , Swiderska‐Syn M , *et al* Pleiotrophin regulates the ductular reaction by controlling the migration of cells in liver progenitor niches. Gut. 2016; 65: 683–92. 25596181 10.1136/gutjnl-2014-308176 PMC4504836

[24] Ochiai K , Muramatsu H , Yamamoto S , *et al* The role of midkine and pleiotrophin in liver regeneration. Liver Int. 2004; 24: 484–91. 15482347 10.1111/j.1478-3231.2004.0990.x

[25] Antoine M , Tag CG , Wirz W , *et al* Upregulation of pleiotrophin expression in rat hepatic stellate cells by PDGF and hypoxia: implications for its role in experimental biliary liver fibrogenesis. Biochem Biophys Res Commun. 2005; 337: 1153–64. 16226713 10.1016/j.bbrc.2005.09.173

[26] Berndt C , Casaroli‐Marano RP , Vilaró S , *et al* Cloning and characterization of human syndecan‐3. J Cell Biochem. 2001; 82: 246–59. 11527150 10.1002/jcb.1119

[27] Asahina K , Sato H , Yamasaki C , *et al* Pleiotrophin/heparin‐binding growth‐associated molecule as a mitogen of rat hepatocytes and its role in regeneration and development of liver. Am J Pathol. 2002; 160: 2191–205. 12057922 10.1016/S0002-9440(10)61167-4 PMC1850835

[28] Leonova EI , Galzitskaya OV . Role of Syndecans in Lipid Metabolism and Human Diseases. Adv Exp Med Biol. 2015; 855: 241–58. 26149933 10.1007/978-3-319-17344-3_10

[29] Marzioni D , Lorenzi T , Mazzucchelli R , *et al* Expression of basic fibroblast growth factor, its receptors and syndecans in bladder cancer. Int J Immunopathol Pharmacol. 2009; 22: 627–38. 19822079 10.1177/039463200902200308

[30] Yao J , Hu XF , Feng XS , *et al* Pleiotrophin promotes perineural invasion in pancreatic cancer. World J Gastroenterol. 2013; 19: 6555–8. 24151381 10.3748/wjg.v19.i39.6555 PMC3801368

[31] Strader AD , Reizes O , Woods SC , *et al* Mice lacking the syndecan‐3 gene are resistant to diet‐induced obesity. J Clin Invest. 2004; 114: 1354–60. 15520868 10.1172/JCI20631 PMC524223

[32] Lee J , Homma T , Kurahashi T , *et al* Oxidative stress triggers lipid droplet accumulation in primary cultured hepatocytes by activating fatty acid synthesis. Biochem Biophys Res Commun. 2015; 464: 229–35. 26116535 10.1016/j.bbrc.2015.06.121

[33] Pais R , Rusu E , Zilisteanu D , *et al* Prevalence of steatosis and insulin resistance in patients with chronic hepatitis B compared with chronic hepatitis C and non‐alcoholic fatty liver disease. Eur J Intern Med. 2015; 26: 30–6. 25553983 10.1016/j.ejim.2014.12.001

[34] Liu L , Hu X , Cai GY , *et al* High glucose‐induced hypertrophy of mesangial cells is reversed by connexin43 overexpression *via* PTEN/Akt/mTOR signaling. Nephrol Dial Transplant. 2012; 27: 90–100. 21633095 10.1093/ndt/gfr265

[35] Hao J , Liu S , Zhao S , *et al* PI3K/Akt pathway mediates high glucose‐induced lipogenesis and extracellular matrix accumulation in HKC cells through regulation of SREBP‐1 and TGF‐β1. Histochem Cell Biol. 2011; 135: 173–81. 21240525 10.1007/s00418-011-0777-3

[36] Gorgani‐Firuzjaee S , Meshkani R . SH2 domain‐containing inositol 5‐phosphatase (SHIP2) inhibition ameliorates high glucose‐induced de‐novo lipogenesis and VLDL production through regulating AMPK/mTOR/SREBP1 pathway and ROS production in HepG2 cells. Free Radic Biol Med. 2015; 89: 679–89. 26456051 10.1016/j.freeradbiomed.2015.10.036

[37] Huang K , Huang J , Chen C , *et al* AP‐1 regulates sphingosine kinase 1 expression in a positive feedback manner in glomerular mesangial cells exposed to high glucose. Cell Signal. 2014; 26: 629–38. 24342046 10.1016/j.cellsig.2013.12.002

[38] Zhou G , Su X , Ma J , *et al* Pioglitazone inhibits high glucose‐induced synthesis of extracellular matrix by NF‐κB and AP‐1 pathways in rat peritoneal mesothelial cells. Mol Med Rep. 2013; 7: 1336–42. 23404530 10.3892/mmr.2013.1309

[39] Poimenidi E , Hatziapostolou M , Papadimitriou E . Serum stimulates Pleiotrophin gene expression in an AP‐1‐dependent manner in human endothelial and glioblastoma cells. Anticancer Res. 2009; 29: 349–54. 19331172

[40] Liu J , Wu X , Liu Y , *et al* High‐glucose‐based peritoneal dialysis solution induces the upregulation of VEGF expression in human peritoneal mesothelial cells: the role of pleiotrophin. Int J Mol Med. 2013; 32: 1150–8. 24042838 10.3892/ijmm.2013.1491

[41] Polytarchou C , Hatziapostolou M , Papadimitriou E . Hydrogen peroxide stimulates proliferation and migration of human prostate cancer cells through activation of activator protein‐1 and up‐regulation of the heparin affin regulatory peptide gene. J Biol Chem. 2005; 280: 40428–35. 16199533 10.1074/jbc.M505120200

[42] Li YS , Hoffman RM , Le Beau MM , *et al* Characterization of the human pleiotrophin gene. Promoter region and chromosomal localization. J Biol Chem. 1992; 267: 26011–6. 1464612

[43] Kim JH , Sinn DH , Gwak GY , *et al* Insulin resistance assessment is useful in risk stratification of hepatocellular carcinoma in chronic hepatitis B patients. J Gastroenterol Hepatol. 2016 Nov 11. doi: 10.1111/jgh.13647. 10.1111/jgh.13647 27862289

[44] Barthel SR , Medvedev R , Heinrich T , *et al* Hepatitis B virus inhibits insulin receptor signaling and impairs liver regeneration *via* intracellular retention of the insulin receptor. Cell Mol Life Sci. 2016; 73: 4121–40. 27155659 10.1007/s00018-016-2259-1 PMC11108314

[45] Yilmaz B , Koklu S , Buyukbayram H , *et al* Chronic hepatitis B associated with hepatic steatosis, insulin resistance, necroinflammation and fibrosis. Afr Health Sci. 2015; 15: 714–8. 26957957 10.4314/ahs.v15i3.3 PMC4765474

[46] Guo CH , Sun TT , Weng XD , *et al* The investigation of glucose metabolism and insulin secretion in subjects of chronic hepatitis B with cirrhosis. Int J Clin Exp Pathol. 2015; 8: 13381–6. 26722544 PMC4680489

[47] Kim S , Lee Y , Koo JS . Differential expression of lipid metabolism‐related proteins in different breast cancer subtypes. PLoS One. 2015; 10: e0119473. 25751270 10.1371/journal.pone.0119473 PMC4353724

[48] Jin X , Zhang KJ , Guo X , *et al* Fatty acid synthesis pathway genetic variants and clinical outcome of non‐small cell lung cancer patients after surgery. Asian Pac J Cancer Prev. 2014; 15: 7097–103. 25227797 10.7314/apjcp.2014.15.17.7097

[49] Williams MD , Zhang X , Park JJ , *et al* Characterizing metabolic changes in human colorectal cancer. Anal Bioanal Chem. 2015; 407: 4581–95. 25943258 10.1007/s00216-015-8662-x

[50] Uehara H , Takahashi T , Oha M , *et al* Exogenous fatty acid binding protein 4 promotes human prostate cancer cell progression. Int J Cancer. 2014; 135: 2558–68. 24740818 10.1002/ijc.28903

[51] Gu D , Yu B , Zhao C , *et al* The effect of pleiotrophin signaling on adipogenesis. FEBS Lett. 2007; 581: 382–8. 17239862 10.1016/j.febslet.2006.12.043

[52] Yi C , Xie WD , Li F , *et al* MiR‐143 enhances adipogenic differentiation of 3T3‐L1 cells through targeting the coding region of mouse pleiotrophin. FEBS Lett. 2011; 585: 3303–9. 21945314 10.1016/j.febslet.2011.09.015

[53] Zou L , Chai J , Gao Y , *et al* Down‐regulated PLAC8 promotes hepatocellular carcinoma cell proliferation by enhancing PI3K/Akt/GSK3β/Wnt/β‐catenin signaling. Biomed Pharmacother. 2016; 84: 139–46. 27643556 10.1016/j.biopha.2016.09.015

[54] Vilchez V , Turcios L , Marti F , *et al* Targeting Wnt/β‐catenin pathway in hepatocellular carcinoma treatment. World J Gastroenterol. 2016; 22: 823–32. 26811628 10.3748/wjg.v22.i2.823 PMC4716080

[55] Jeon M , Rahman N , Kim YS . Wnt/β‐catenin signaling plays a distinct role in methyl gallate‐mediated inhibition of adipogenesis. Biochem Biophys Res Commun. 2016; 479: 22–7. 27592552 10.1016/j.bbrc.2016.08.178

[56] Chung SS , Lee JS , Kim M , *et al* Regulation of Wnt/β‐catenin signaling by CCAAT/enhancer binding protein β during adipogenesis. Obesity (Silver Spring). 2012; 20: 482–7. 21760632 10.1038/oby.2011.212

