# Supplementary figures and images for: Pleiotrophin, a target of miR‐384, promotes proliferation, metastasis and lipogenesis in HBV‐related hepatocellular carcinoma
Source: J Cell Mol Med. 2017 May 30;21(11):3023–43. doi: 10.1111/jcmm.13213 (PMC5661149; doi:10.1111/jcmm.13213)

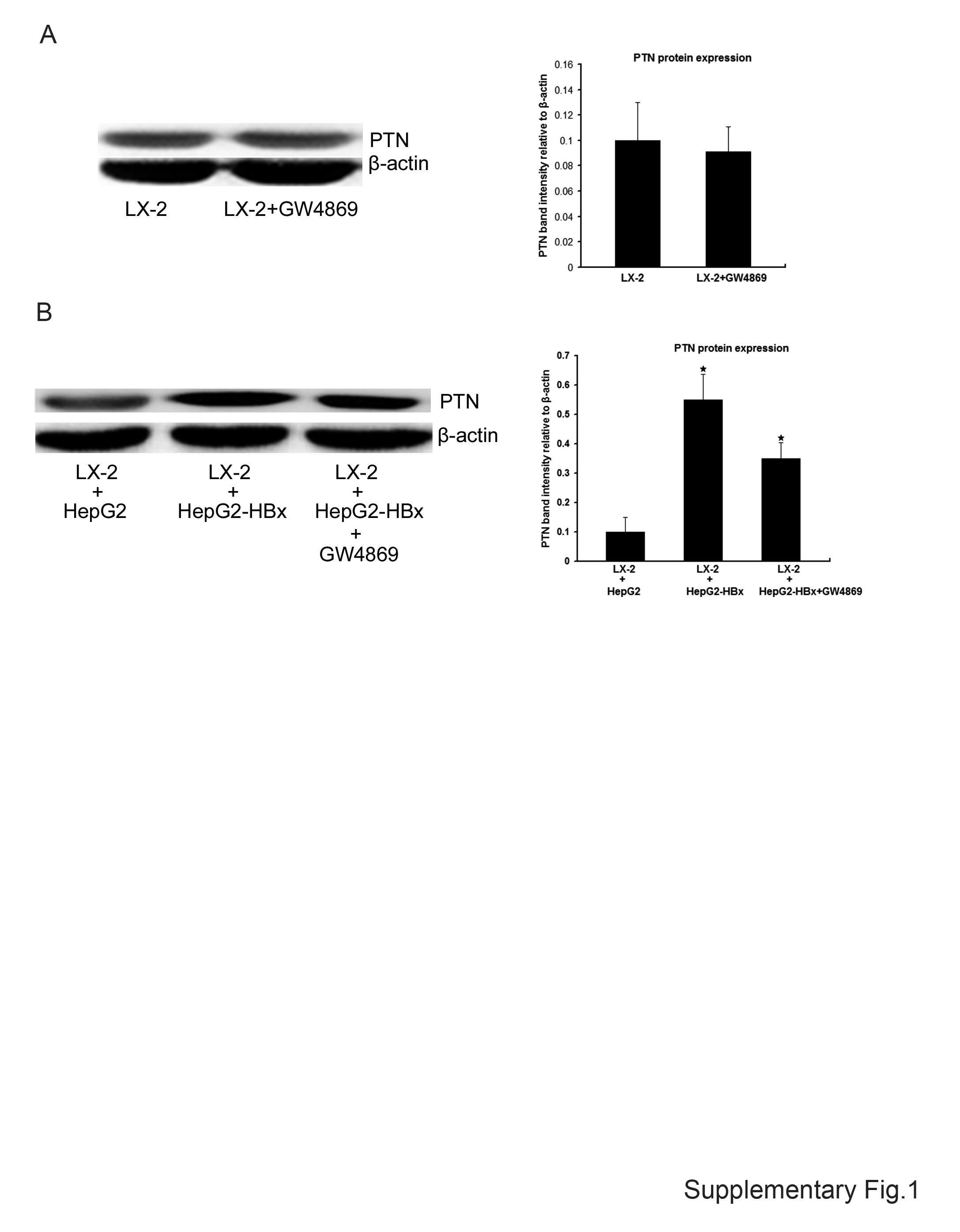

Supplement: Supplementary file 1 — Figure S1 The inhibition of exosome secretion through GW4869 could reduce the PTN expression in LX‐2 co‐cultured with HepG2‐HBx. [file JCMM-21-3023-s001.tif]
